# Botulinum Toxin Type A for the Prevention of Migraines: An Umbrella Review of Systematic Reviews

**DOI:** 10.3390/toxins18010033

**Published:** 2026-01-09

**Authors:** Goli Chamani, Hajer Jasim, Ava Minston, Marlon Ferreira Dias, Rodrigo Lorenzi Poluha, Daniela A. Godoi Gonçalves, Maria Christidis, Essam Ahmed Al-Moraissi, Nikolaos Christidis, Giancarlo De la Torre Canales, Malin Ernberg

**Affiliations:** 1 Division of Oral Rehabilitation, Department of Dental Medicine, Karolinska Institutet, SE-14104 Huddinge, Sweden; goli.chamani@ki.se (G.C.); hajer.jasim@ki.se (H.J.); ava.minston@ki.se (A.M.); marlon.dias@unesp.br (M.F.D.); nikolaos.christidis@ki.se (N.C.); giancarlo.de.la.torre.canales@ki.se (G.D.l.T.C.); 2 Department of Orofacial Pain and Jaw Function, Public Dental Services, Folktandvården Stockholm, Eastmaninstitutet, SE-10231 Stockholm, Sweden; 3 Department of Dental Materials and Prosthodontics, School of Dentistry, São Paulo State University (UNESP), Araraquara 14801-903, Brazil; daniela.g.goncalves@unesp.br; 4 Department of Dentistry, State University of Maringa (UEM), Maringá 87083-170, PR, Brazil; rodrigopoluha@gmail.com; 5 Department of Nursing Science, Sophiahemmet University, SE-114 86 Stockholm, Sweden; maria.christidis@shh.se; 6 Department of Neurobiology, Care Sciences and Society, Karolinska Institutet, SE-14183 Huddinge, Sweden; 7 Department of Oral and Maxillofacial Surgery, Faculty of Dentistry, Thamar University, Thamar 009676, Yemen; dressamalmoraissi@gmail.com; 8 Egas Moniz Center for Interdisciplinary Research (CiiEM), Egas Moniz School of Health & Science, Caparica, 2829-511 Almada, Portugal

**Keywords:** botulinum toxin type A, chronic migraine, umbrella review, quality of life, CGRP monoclonal antibodies, preventive therapy

## Abstract

Botulinum toxin type A (BoNT-A) is an established preventive therapy for chronic migraines; however, uncertainty remains regarding its comparative efficacy and safety. Thus, we aimed to summarize current evidence from high-quality systematic reviews of the therapeutic effects of BoNT-A in migraine management. An umbrella review was conducted following PRISMA guidelines and registered in PROSPERO. High-quality systematic reviews with meta-analysis evaluating BoNT-A efficacy were identified through five databases up to August 2024. Primary outcomes included monthly headache frequency and severity. Methodological quality and risk of bias were assessed using the umbrella review checklist. Fourteen articles were included. Overall, quantitative evidence indicated favorable effects of BoNT-A compared with placebo for chronic migraines, across headache frequency, headache severity, and acute medication use, but less efficacy than topiramate and the CGRP monoclonal antibodies (CGRPmAbs) galcanezumab and fremanezumab. Though the adverse events were frequent, BoNT-A was generally well-tolerated. Comparative data suggested superior tolerability versus topiramate and a safety profile like CGRPmAbs. Although botulinum toxin type A is widely used as a preventive treatment for chronic migraines, the available evidence supports its efficacy at a moderate level. Further head-to-head and long-term analyses are needed to clarify its comparative role alongside newer biologic treatments.

## 1. Introduction

Migraines are a prevalent chronic neurological disorder associated with substantial disability, diminished quality of life, and reduced work capacity among affected individuals [1]. They are characterized by a moderate or severe unilateral, pulsating headache that is aggravated by physical activity and associated with nausea and/or photophobia, phonophobia, and cutaneous allodynia [2,3]. Globally, migraines rank as the third most common disease, affecting approximately one in seven individuals (14.8% to 18.5% in North America and Europe) [1], with women disproportionately affected and more likely to report migraine-related disability [4,5]. Moreover, migraines are labelled among the most disabling diseases worldwide. The Global Burden of Disease 2021 showed that migraines are the second most disabling neurological disease among adults, right after strokes [6].

Two main subtypes of migraines are recognized: migraines with aura and migraines without aura. A migraine is classified as episodic when attacks occur fewer than 15 days per month, and as chronic when attacks occur on at least 15 days per month for three or more consecutive months [3]. As no curative treatments currently exist, therapeutic strategies focus primarily on prevention and symptom control [5,7]. Oral preventive therapies, including antihypertensives, antidepressants, and anticonvulsants, are commonly prescribed for chronic migraines [2,8,9,10]. However, their efficacy remains limited, and treatment discontinuation frequently occurs due to poor tolerability, inadequate efficacy, or non-adherence [9,10,11,12].

Botulinum toxin type A (BoNT-A), a neurotoxin produced by Clostridium botulinum, has emerged as a preventive therapy for both episodic and chronic migraines [8]. Initially proposed by Binder in 1998 for headache management following cosmetic injections [13], BoNT-A exerts both paralytic effects at the neuromuscular junction and antinociceptive effects via peripheral and central mechanisms [13,14]. It blocks acetylcholine release at the neuromuscular junction, thereby reducing peripheral nociceptive signaling and central sensitization [13,14]. Evidence from animal and human studies indicates that BoNT-A also inhibits the release of pain-related neuropeptides, such as substance P (SP) and calcitonin gene-related peptide (CGRP), modulates spinal cord neuronal activity, and may undergo retrograde axonal transport to the central nervous system, thereby influencing central pain processing [15]. Inflammatory mediators play a critical role in sensitizing peripheral pain receptors, leading to central sensitization and persistent pain [16]. Advances in migraine pathophysiology over the past decade highlight the central role of CGRP, released within the trigeminovascular system, which promotes neurogenic inflammation and vasodilation [17]. Preclinical studies demonstrate CGRP activity in both the central and peripheral nervous systems, targeting mast cells, blood vessels, glial cells, trigeminal afferents, and satellite glial cells [18,19]. BoNT-A interferes with these inflammatory pathways, suggesting both peripheral and central mechanisms of action [14]. In addition, BoNT-A may directly inhibit central pain transmission, possibly through mechanisms resembling those of endogenous opioids [20,21].

In 2010, the U.S. Food and Drug Administration approved BoNT-A for the preventive treatment of chronic migraines. Clinical trials consistently report its efficacy and tolerability, demonstrating reductions in headache frequency and severity, decreased reliance on symptomatic medications, and improved quality of life [22,23]. Systematic reviews (SRs) and network meta-analyses suggest that BoNT-A offers one of the most favorable efficacy and safety profiles among preventive therapies, with potentially greater patient acceptance compared to other agents, although direct comparative evidence remains limited [24,25].

Despite robust clinical evidence, several uncertainties persist regarding BoNT-A therapy, including its peripheral and central mechanisms, optimal injection patterns, long-term effects on migraine chronification, efficacy in episodic migraines and other headache disorders, and the potential benefits of shorter dosing intervals [10,22]. Moreover, the absence of standardized treatment protocols and definitive conclusions regarding their overall efficacy and safety underscores the need for a comprehensive synthesis. Accordingly, the present study aimed to conduct an umbrella review with meta-analysis of high-quality SRs evaluating the therapeutic effects of BoNT-A in migraine management.

## 2. Results

### 2.1. Study Selection

**Figure 1:**
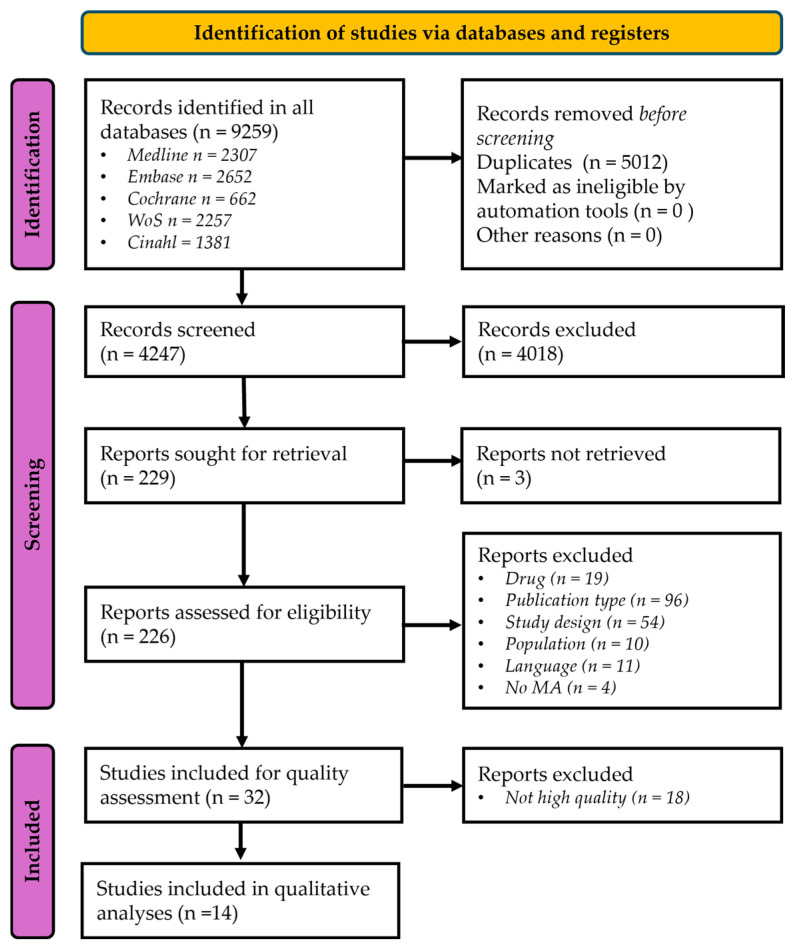
The PRISMA flow-chart of the database search strategy. MA: Meta-analysis.

Across all databases, 9258 records were identified. After removing 5011 duplicates, 4247 titles/abstracts were screened, and 4018 were excluded for ineligibility. A total of 229 articles were sought for full-text review; a total of 3 could not be accessed. Following full-text assessment, 32 articles were included and assessed for methodological quality/risk-of-bias appraisal. Eighteen articles were not deemed of high quality and excluded (Tables S1 and S2). Thus, 14 studies met all eligibility criteria and were included in the synthesis [7,10,13,14,26,27,28,29,30,31,32,33,34,35] (Figure 1).

### 2.2. Study Characteristics

The 14 included meta-analyses [7,14,26,27,28,29,30,31,32,33,34,35] were published between 2009 and 2024. Most reviews evaluated the efficacy and safety of BoNT-A for preventing or reducing the severity of episodic and chronic migraines; sample sizes ranged from 491 to 13,302 participants. Adults predominated, with comparatively fewer pediatric/adolescent data [10,28,35]. Study characteristics are summarized in Table S3.

The included studies comprised a total of 99 different primary studies that were included in between one and nine of the meta-analyses. Thirty-nine of the primary studies were included in more than one SR (Table S4).

### 2.3. Doses and Injection Protocols

Across included SRs, most original studies employed the PREEMPT regimen of 155 U onabotulinumtoxinA (up to 195 Units (U) with a “follow-the-pain” approach) injected at 31–39 sites every 12 weeks [7,10,13,14,31,32]. Lower doses (25–100 U) were used only in earlier or localized protocols and generally demonstrated reduced efficacy [26,27]. Observational and registry studies included in SRs also followed the PREEMPT dosing pattern with repeated 12-week cycles [30,33]. Alternative formulations (abobotulinumtoxinA, incobotulinumtoxinA) were seldom used [7].

### 2.4. Headache Frequency

Table 1 presents data from studies concerning headache frequency and severity. Across the included SRs and meta-analyses, BoNT-A was evaluated against several comparator types: (1) the patient’s own baseline status prior to BoNT-A administration [30,33,35]; (2) placebo injections in randomized trials [7,10,13,14,26,27,31,32,34]; (3) active preventive treatments, including topiramate, acupuncture, and CGRP monoclonal antibodies (CGRPmAbs) such as fremanezumab, galcanezumab, and eptinezumab, using direct or network meta-analytic comparisons [10,29,31,32,34]; and (4) alternative BoNT-A dosing regimens [7]. Follow-up durations ranged from 1 to 12 months. The outcomes used in the different SRs for headache frequency varied and included a change in the number of monthly migraine days/episodes, a change in the number of monthly headache days/episodes, and a ≥50% reduction in migraine/headache days/episodes. Many SRs included several outcomes to capture headache frequency. Though most of them included only one primary frequency outcome, four SRs included two [10,14,29,31].

Results for monthly headache frequency varied across SRs. Generally, BoNT-A was associated with weak and mostly insignificant reductions in episodic migraines [7,13,14,26,27,32], but most studies in chronic migraines showed significant improvement [7,13,27,28,30,32,33,35]. The four SRs that included two outcome measures of frequency showed different effects [10,14,29,31].

Compared to placebo, BoNT-A produced short-term reductions (2–3 months) in chronic migraine frequency [13,29,31], with benefits maintained at 6 months in several analyses [28,30]. The Cochrane review [7] and later syntheses [32,33] confirmed that this reduction persists over repeated injection cycles up to 9–12 months, supporting the durable preventive efficacy of BoNT-A for chronic migraines. However, BoNT-A was less effective for the prevention of chronic migraines than topiramate, galcanezumab, and fremanezumab [29,31].

Evidence for the efficacy of BoNT-A in episodic migraines remains limited and less consistent than for chronic migraines. Meta-analyses including participants with episodic migraines reported no significant reduction in monthly migraine days compared with placebo, and effect estimates were small and heterogeneous [7,13,14]. In pooled analyses restricted to episodic migraines, BoNT-A failed to achieve clinically meaningful improvements in headache frequency or intensity [7,14]. These findings align with the PREEMPT trials, which primarily demonstrated benefit in chronic rather than episodic subgroups [7]. Collectively, current evidence suggests that BoNT-A is not consistently effective for episodic migraine prevention, and its therapeutic benefit appears confined to patients with chronic migraines [7,10,13,14].

### 2.5. Headache Severity

Evidence on migraine headache severity outcomes, such as the Visual Analog Scale (VAS), showed considerable heterogeneity across studies (Table 2). Most studies compared changes after BoNT-A vs. baseline.

Significant reductions in migraine intensity (VAS) with BoNT-A compared to baseline or placebo were reported in all four meta-analyses [7,14,30,35]. Follow-up evidence indicates that BoNT-A significantly reduces headache severity over time. In one review that included adults with chronic migraines, pain intensity improved at six months after treatment [30]. Randomized trials included in two SRs demonstrated meaningful reductions in severity compared with placebo after approximately 12 weeks [7,14]. Similar sustained improvements were also reported in pediatric and adolescent patients after multiple treatment cycles [35].

### 2.6. Quality of Life

Table 3 presents data for quality-of-life outcomes. Overall, only a few randomized controlled trials have assessed quality-of-life outcomes, and findings remain mixed and methodologically heterogeneous. The effects were assessed through instruments reflecting both functional and psychological dimensions of well-being. Consistent improvements in functional outcomes were reported using the Headache Impact Test (HIT-6) and Migraine Disability Assessment Score (MIDAS) when compared to placebo but not when compared to other treatment modalities. For the Migraine-Specific Questionnaire (MSQ), there was an improvement compared to baseline. This indicates a reduced headache-related impact and disability after treatment [13,14,29,30], when compared to placebo and to baseline. However, other analyses reported negligible or inconsistent effects of BoNT-A on HIT-6 scores [14]. Limited comparative evidence also suggests that alternative treatments, such as fremanezumab, may yield greater reductions in headache impact than BoNT-A [31].

Meaningful enhancements in migraine-specific quality of life were noted during six-to-twelve-month follow-up periods [29,30]. Pooled analyses also showed overall quality-of-life improvements compared with placebo [13], although one meta-analysis reported a non-significant change in HIT-6 [14]. Regarding psychological well-being, significant reductions were found in the Beck Depression Inventory (BDI and BDI-II) and Patient Health Questionnaire-9 (PHQ-9) scores, suggesting improvement in depressive symptoms following therapy [30]. Taken together, these findings support that BoNT-A exerts favorable effects on both functional ability and emotional health, contributing to an overall enhancement in quality of life [10,13,14,29,30,33], but not in comparison to other treatment modalities.

### 2.7. Use of Rescue Medication and Adverse Events

Table 4 presents data for the use of rescue medication and adverse events. Several meta-analyses evaluated the impact of BoNT-A on the use of acute or rescue migraine medications, generally showing a reduction in consumption frequency among patients with chronic migraine [7,13,29,33]. This reduction in acute medication use paralleled the decrease in monthly migraine or headache days, suggesting that preventive treatment with BoNT-A lowers reliance on rescue therapy [7,29,33]. However, some analyses described modest or non-significant changes, particularly in shorter-duration studies or smaller samples [10,14]. Overall, the available evidence supports an association between BoNT-A and a clinically meaningful reduction in acute medication use in chronic migraine, which may help limit medication overuse and improve long-term management [7,10,13,14,29,30,33].

Across the included SRs, BoNT-A demonstrated an adequate safety and tolerability profile [7,13,33]. Reported adverse events were predominantly mild and transient, most commonly including injection-site pain, neck pain, eyelid or eyebrow ptosis, and localized muscle weakness [7,13,30,33]. Serious or systemic reactions were rare [7,14,29]. Comparative analyses indicated fewer treatment-related discontinuations with BoNT-A than with topiramate, and an overall safety profile like that of CGRPmAbs [10,29,33]. No new safety signals emerged with longer follow-up, and cumulative exposure did not appear to increase risk [7,13,29,30,33]. Taken together, the evidence supports BoNT-A as a reasonably well-tolerated preventive option with largely manageable adverse effects [7,10,13,14,29,30,33].

### 2.8. Cost-Effectiveness

Cost-effectiveness was not assessed in any of the included SRs.

### 2.9. Synthesis Across Outcomes

Table 5 presents results from the synthesis across outcomes. Overall, quantitative evidence indicates negligible-to-favorable effects of BoNT-A compared with placebo or active comparators across monthly migraine days, headache severity, and acute medication use. Some meta-analyses demonstrated significant benefits of BoNT-A over placebo or when compared to baseline [7,13,14,27,31,33], particularly in reducing monthly migraine days, whereas findings for headache severity and medication use were more variable. Subgroup and time-point analyses highlighted heterogeneity in treatment response and suggested that therapeutic effects may depend on migraine subtype, follow-up duration, and baseline episode frequency [26,28]. Comparative data remain limited; one network meta-analysis indicated that BoNT-A and topiramate achieved broadly comparable short-term efficacy [29], while another showed that CGRPmAbs often yielded greater improvements in selected outcomes [31]. Overall, the totality of evidence does not show any unanimous agreement on the efficacy and tolerability of BoNT-A. However, it suggests that BoNT-A can be used as a complementary preventive therapy for chronic migraines.

## 3. Discussion

Based on a synthesis of the included high-quality SRs, there is no unanimous consensus on the effectiveness of BoNT-A in migraine management. The results indicate that the benefit of this treatment compared to placebo is consistent in chronic migraines, with a significant pain reduction after BoNT-A treatment [7,14,30,35]. However, the efficacy of BoNT-A was not superior to acupuncture, topiramate, or CGRPmAbs [10,29,31]. Findings concerning the effectiveness of BoNT-A in episodic migraines were typically non-significant. Our review also showed that, despite a high risk of adverse events, BoNT-A is well-tolerated, as adverse effects were generally mild and transient and comparable in frequency to those observed with placebo and other treatments.

Most SRs showed a superior effect for headache frequency (reduction in monthly migraine/headache days, monthly migraine/headache episodes, or ≥50% reduction in monthly days/episodes) compared to placebo in chronic migraines [7,13,27,28,30,32,33,35]. However, four SRs included two primary outcomes for frequency, which showed different results [10,14,29,31]. For three of them, this can perhaps be explained by fewer studies and patients with non-significant improvement [10,29,31] and thus with a greater risk of type I error. For example, Zheng [29] included only two studies with 110 participants for the reduction in monthly migraine days, but nine studies with 948 participants for the reduction in monthly headache days. Migraine frequency, when used as a secondary outcome in chronic migraines, showed more negative results [7,10,29,31], but more often included a reduction in episodes. This number does not capture the full migraine burden, as it would weigh unrelenting 3-day-long attacks and those that resolve in just one day or less, equivalently, despite the highly relevant difference for patients. Similarly, non-significant pooled effect sizes may be due to the low number of included studies reporting on the specific outcome.

BoNT-A is clinically used worldwide for the treatment of chronic migraines, due to positive results of the pooled analysis of two multicenter studies in the Phase III Research Evaluating Migraine Prophylaxis Therapy (PREEMPT) clinical program [36]. The results of this analysis demonstrated reductions in migraine days and pain intensity, findings that are consistent with our data. These beneficial effects are primarily attributed to the well-established pain-relieving properties of BoNT-A, demonstrated in animal studies through various mechanisms underlying its antinociceptive action [37]. BoNT-A inhibits the release of CGRP, SP, serotonin, glutamate, gamma aminobutyric acid, noradrenaline, dopamine, and glycine, which are neurotransmitters associated with primary headache disorders [38]. Moreover, BoNT-A suppresses the surface expression of nociceptive transient receptor potential channels associated with headache disorders [39,40,41,42], thereby limiting the release of CGRP and SP and, consequently, reducing vasodilation and inflammatory mediators’ release [43]. Due to the ability of BoNT-A to undergo axonal transport, animal research supports the modulation of these neurotransmitters in peripheral nerve terminals (reduced peripheral sensitization) and in the central nervous system (reduced central sensitization) [37]. Despite the fact that the influence of extracranial intramuscular BoNT-A injections on intracranial nociceptive pathways remains incompletely understood, their dual action on peripheral and central sensitization makes them a proper candidate for the treatment of migraines. The moderate effect of BoNT-A compared with placebo observed in this review suggests that these mechanisms may be the primary drivers in this headache disorder.

Moreover, even though most of the included SRs reported that trials adhered to PREEMPT 1 and 2 protocols (155 and 195 units, respectively) [30], other reported doses range from 16 to 225 units, with lower doses reporting smaller effects [32]. It is important to note that the PREEMPT protocol is the only validated protocol for chronic migraines, in which from 155 to 195 units are injected into eight regions distributed in the face, head, and neck muscles (corrugator, procerus, frontalis, temporalis, occipitalis, cervical paraspinal, and trapezius) every 3 months up to five times. Interestingly, most of the included studies reported a beneficial effect just after 3 months of treatment, which raises questions about the clinical efficacy, doses, and follow-up times of this protocol, as well as the cost-effectiveness of BoNT-A treatment. Unfortunately, none of the included SRs had cost-effectiveness as an outcome, so this could not be evaluated. However, previous studies have shown that the cost to avoid headache episodes was lower for BoNT-A than for CGRPmAbs [44,45,46]. Thus, in line with another umbrella review from our group on BoNT-A for temporomandibular disorders [47], we recommend that clinicians limit its application to chronic migraine cases in which standard treatments do not relieve pain significantly.

Although the included instruments assess different domains, they collectively represent the broader construct of health-related quality of life. HIT-6, MIDAS, and MSQ primarily evaluate the functional and activity limitations associated with migraines, while BDI and PHQ-9 capture their psychological burden [7,13,14,29,30]. Integrating these measures under the category “Quality of life” is consistent with established frameworks of health-related quality of life, which encompass both physical and mental components of disease impact [33]. Within this multidimensional framework, the improvements observed across functional and emotional outcomes indicate that BoNT-A provides a meaningful benefit extending beyond symptom control to overall patient well-being, when compared to placebo, although not compared with other treatment options [7,10,13,14,29,30,33].

Finally, our findings also indicate that BoNT-A presented a well-tolerated safety profile across the included SRs, displaying transient minor or mild adverse events, which were commonly related to the injection procedure, transient ptosis, and localized muscle weakness [7,29,34]. In addition, we found that, compared with topiramate and CGRPmAbs, BoNT-A is generally better tolerated and has a similar profile, respectively [10,31]. It is suggested that only trained clinicians with knowledge of the topographic anatomy of the face, head, and neck areas should perform injections of BoNT-A.

This umbrella review systematically synthesized evidence from 14 high-quality SRs with meta-analyses, representing the most comprehensive evaluation to date of BoNT-A for migraine prevention. Its broad, time-unrestricted, multi-database search ensured wide coverage of the existing literature and inclusion of heterogeneous populations (primarily adults, with some adolescents), thereby enhancing generalizability. The study provided a multidimensional synthesis across migraine frequency, severity, quality of life, rescue medication use, and adverse events. By integrating findings from previous SRs and meta-analyses, this umbrella review offers a consolidated and accessible overview of the totality of evidence, which can inform clinical decision-making and guideline development. A further methodological strength is the strict inclusion of only high-quality reviews, based on predefined criteria and independent appraisal, which improves the reliability of the conclusions. Notably, some recent network meta-analyses comparing BoNT-A with CGRP-targeted therapies were not included due to methodological limitations according to our appraisal criteria; for instance, Naghdi et al. 2023 [48]. While these studies often report findings broadly consistent with the comparative trends observed in the present review, their exclusion reflects differences in methodological rigor rather than disagreement with their conclusions.

However, several limitations should be acknowledged. The umbrella review’s conclusions are inherently dependent on the quality and reporting of the included SRs and their primary studies. Substantial between-study heterogeneity was observed across populations, study designs, interventions, and outcome measures, which complicates pooled interpretation. Overlap of primary trials among included reviews introduces the potential risk of double-counting and biasing overall estimates. Indeed, more than one-third of the primary studies were included in more than one of the meta-analyses, and several primary studies were included in seven to nine of the meta-analyses. Moreover, some SRs incompletely reported key outcomes such as migraine severity, quality of life, and adverse events, reducing analytic precision. Another limitation is that relatively few studies addressed medication use, quality of life, or cost-effectiveness, limiting the robustness of conclusions in these domains. Further, several potentially informative systematic reviews were excluded due to insufficient methodological quality, which was most commonly related to incomplete assessment of publication bias or small-study effects. This strict inclusion strategy may have reduced the breadth of the evidence base. However, this strict cut-off level for inclusion was intentional and aligned with the primary aim of synthesizing only high-quality evidence. In umbrella reviews, inclusion of methodologically weaker reviews may lead to cumulative bias, whereby limitations in primary syntheses propagate and potentially distort higher-level conclusions. Future work could explore sensitivity analyses that incorporate lower-quality reviews to evaluate the robustness of findings derived from high-quality evidence alone.

Finally, while this review is novel as the first umbrella review addressing the effect of Botulinum toxin type A for the prevention of migraines, its incremental contribution may be limited by the fact that BoNT-A is already widely approved, guideline-supported, and well-accepted as an effective and low-risk treatment for chronic migraines.

## 4. Conclusions

This umbrella review provides the most comprehensive synthesis to date of the evidence regarding BoNT-A for the prevention of migraines. Overall, across 14 high-quality SRs with meta-analyses, the totality of evidence showed favorable effects of BoNT-A compared with placebo for chronic migraines but not for episodic migraines, and no superior effect compared to active comparators across monthly migraine/headache frequency, severity, and acute medication use. Its safety profile was reasonable, with predominantly mild and transient adverse effects and better tolerability than topiramate. Evidence on migraine severity, quality of life, and medication use was more limited and heterogeneous. Comparative data indicate that BoNT-A may achieve clinically meaningful benefits in chronic migraines, but that emerging CGRPmAbs, such as fremanezumab, may offer greater efficacy.

Considering the moderate evidence for its efficacy for migraine prophylaxis, BoNT-A can be recommended as a complementary preventive therapy for patients with chronic migraines who are unresponsive to or intolerant of oral preventives or biologics. Future research should prioritize direct head-to-head randomized trials comparing BoNT-A with CGRP-targeted therapies, evaluate potential combination regimens, and include long-term assessments of cost-effectiveness and patient-reported outcomes to better define its comparative and complementary role in migraine prevention.

## 5. Materials and Methods

### 5.1. Protocol

This umbrella review followed the Preferred Reporting Items for Systematic Reviews and Meta-Analyses (PRISMA) guidelines (https://www.prisma-statement.org/ access date 5 January 2026) (Table S5) and was registered in the International Prospective Register of Systematic Reviews (PROSPERO) database (https://www.crd.york.ac.uk/prospero/ access date 30 December 2024), registration ID: CRD42024542985. An amendment was made with an adjustment of the author list and outcomes.

### 5.2. Inclusion Criteria

Study eligibility was defined using the Population, Intervention, Comparators, Outcomes, Timing, and Study design (PICOTS) framework: Population (P): Individuals with migraine. Intervention (I): Treatment with botulinum toxin type A (BoNT-A). Comparators (C): No treatment, placebo, or other active treatments. Outcomes (O): Monthly migraine frequency (migraine days, migraine episodes, ≥50% reduction in migraine days/episodes), migraine severity (VAS), headache impact (HIT-6, MIDAS, BDI, PHQ-9), quality of life (HIT-6, MIDAS, BDI combined, and MSQ), use of rescue medications, adverse events, and cost-effectiveness. Timing (T): Short-, intermediate-, or long-term outcomes. Study design (S): Only SRs with a meta-analysis reporting at least one predefined outcome. Primary outcomes were migraine frequency and severity, while headache impact, quality of life, use of rescue medications, adverse events, and cost-effectiveness served as secondary outcomes.

### 5.3. Exclusion Criteria

Editorials, letters, legal cases, interviews, case series, duplicates, observational studies, cross-sectional studies, and case–control studies were excluded. We further excluded publications not written in English, the Scandinavian languages, Portuguese, Spanish, or Greek; SRs rated as low-quality according to the critical appraisal checklist; and non-eligible publication types, including editorials, letters, legal cases, interviews, case series, duplicates, and observational designs such as cross-sectional and case–control studies.

### 5.4. Search Strategy

A literature search was performed in the following five databases: Medline (Ovid), Embase (embase.com), Cochrane Library (Wiley), Web of Science (Clarivate Analytics), and Confidence interval (CI)NAHL (EBSCOhost). For detailed search strategies, see Table S6. The last search was conducted on 20 August 2024. The search strategy was developed in Medline (Ovid) in collaboration with librarians at the Karolinska Institutet University Library. For each search concept, Medical Subject Headings (MeSH)-terms and free-text terms were identified. The search was then translated, in part using Polyglot Search Translator [49], into the other databases. Databases were searched from inception, and language restriction was restricted to English, Nordic languages, Portuguese, Spanish, or Greek. The strategies were proof-read by another librarian prior to execution. A snow-ball search was applied to check references and citations of eligible studies from the database searches.

### 5.5. Study Selection

All search results were imported into the Rayyan AI-powered Systematic Review Management Platform (https://www.rayyan.ai accessed on 15 December 2025) for de-duplication and screening [50,51]. Duplicates were removed following Bramer et al.’s method [52], with an additional DOI check for accuracy. Two reviewers (H.J. and A.M.) independently screened titles and abstracts. Any study included by at least one reviewer proceeded to full-text review. Full-text screening was performed in duplicate by the same reviewers, with disagreements resolved by consensus or by a third reviewer (M.E.). The complete search strategies for all databases are available in the Supplementary Material.

### 5.6. Assessment of Methodological Quality and Risk of Bias

Two reviewers (M.C. and R.L.P.) independently assessed methodological quality and risk of bias using the critical appraisal checklist developed by the umbrella review methodology working group [53]. Any disagreement was resolved by a judge (N.C.).

SRs were judged high-quality/low risk of bias if they satisfied all of a pre-specified set of core criteria: clearly defined and appropriate inclusion criteria; a comprehensive, reproducible search strategy with justified information sources; independent critical appraisal performed by at least two reviewers with a predefined process for resolving disagreements; appropriate assessment of small-study effects and publication bias when applicable; and conclusions that were demonstrably supported by the presented data. Additional desirable items, such as detailed reporting of synthesis methods or recommendations for future research, were recorded but did not affect the overall quality rating (Table S1).

The decision to include only systematic reviews meeting all predefined core quality criteria was made prior to study selection and was intended to ensure a high level of methodological rigor across the evidence base. Particularly, appropriate assessment of small-study effects and publication bias was considered essential when applicable, as failure to address these issues may compromise the validity of pooled estimates, even when only randomized controlled trials are included. Reviews that did not fulfill these criteria were, therefore, excluded to minimize risk-of-bias reproduction in this higher-order synthesis.

### 5.7. Data Extraction

A standardized data extraction form was adapted from a previous review [22] and piloted in two studies for consistency. Data extraction was performed independently by two reviewers (M.E. and M.F.D.). Two other reviewers (G.D.l.T.C. and G.C.) resolved any discrepancies. Extracted data included the following: author names, type of SR, publication year, study objectives, diagnostic criteria, patient demographics, interventions and comparators, search dates and databases, included study characteristics, quality assessment tools, synthesis methods, and reported outcomes. For the outcome “quality of life”, data were extracted from studies reporting any measure of health-related quality-of-life or related patient-reported outcomes. This domain was defined broadly to encompass both functional and psychological dimensions of migraine burden. Instruments capturing functional impact, including the MIDAS, HIT-6, and MSQ, were analyzed together with measures assessing psychological morbidity, such as the BDI/BDI-II and the PHQ-9. Grouping these tools under a unified domain of “Quality of life” aligns with the conceptual framework of health-related quality of life, which integrates both physical and mental health aspects of disease experience. When multiple tools were reported in a single meta-analysis, results were summarized narratively and categorized by functional or psychological dimension.

### 5.8. Data Synthesis and Visualization

Evidence synthesis and data validation were undertaken by two reviewers (N.C. and G.D.l.T.C). Findings were organized in structured Tables and visualized using a traffic-light scheme to convey direction and certainty of effects at a glance: green indicated a beneficial effect and/or no adverse events; orange indicated no significant difference and/or mild adverse events; and red indicated a detrimental effect and/or moderate-to-severe adverse events. This presentation was selected to provide a concise overview of efficacy and safety while maintaining transparency.

### 5.9. Meta-Analysis

The included SRs used many different outcomes, analytical methods, and units for the outcomes (mean difference, standardized mean difference, weighted mean difference, relative risk, and odds ratio). This meant that only very few SRs could be combined in each meta-analysis. We therefore refrained from quantitative synthesis of data.

## Figures and Tables

**Table 1:** Effect of BoNT-A treatment on headache frequency.

Author, Year	Outcome Intervention(s)	Number of Studies/ Participants	Results/Findings	Heterogeneity
*Change in monthly migraine days*				
Affatato, 2021 [30]	BoNT-A vs. baseline, 6 months, CM	5/180	MD: −6.70 (95% CI [−9.70 to −3.70]), **p < 0.01**	I^2^ = 100%, Q = 1110, p = 0.00
Herd, 2018 [7]	BoNT-A vs. placebo, 12 weeks, EM	1/418	MD: −0.20 (95% CI [−0.77 to 0.37]), p = 0.490	NA
	BoNT-A vs. placebo, 12 weeks, CM	4/1497	MD: −3.07 (95% CI [−4.73 to −1.41]), **p < 0.001**	I^2^ = 76%, Chi^2^ = 13, p = 0.006
Chen, 2021 [31]	BoNT-A vs. placebo, 12 weeks, CM	NR/110	SMD: 0 (95% CI [−0.43, 0.43]), NS	I^2^ = 43%, Q = 1.7, p = 0.187
	Galcanezumab 120 mg vs. BoNT-A, 12 weeks, CM	NR	SMD: −0.87 (95% CI [−1.50 to −0.24]), **p < 0.05**	
	Fremanezumab 675 mg vs. BoNT-A, 12 weeks, CM	NR	SMD: −0.48 (95% CI [−1.10 to 0.15]), NS	
	Eptinezumab 300 mg vs. BoNT-A, 12 weeks, CM	NR	SMD: −0.38 [95% CI [−1.05 to 0.28]), NS	
	NMA, 12 weeks, CM	4/2452	Galcanezumab 120 mg: P score = 0.92	
			Fremanezumab 675 g: P-score = 0.63	
			Eptinezumab 300 mg: P score = 0.55	
			BoNT-A: P score = 0.17	
Zhao et al., 2024 [10]	BoNT-A vs. placebo, time NR, CM	NR	WMD: −3.88 (95% CI [−0.48 to −7.28]), **p < 0.05**	NR
	BoNT-A vs. topiramate, time NR, CM	NR	WMD: 0.69 (95% CI [−2.97 to 4.35]), NS	
	NMA, time NR, CM	21/6654	BoNT-A: SUCRA = 0.67	
			Topiramate: SUCRA = 0.58	
			Eptinezumab: SUCRA = 0.48	
			Galcanezumab: SUCRA = 0.45	
			Fremanezumab: SUCRA = 0.37	
Zheng, 2020 [29]	BoNT-A vs. placebo, 12 weeks, CM	2/110	SMD: 0.01 (95% CI [−0.38 to 0.41]), NS	I^2^ = 39%
	Topiramate vs. BoNT-A, 12 weeks, CM	NR	SMD: −0.41 (95% CI [−0.94 to 0.12]), NS	
	NMA, 12 weeks, CM	4/295	Topiramate: P score = 0.96	
			BoNT-A: P score = 0.27	
***Change in monthly headache days***				
Lanteri-Minet, 2022 [33]	BoNT-A vs. baseline, 24 weeks, CM	7/2089	MD: −10.64 (95% CI [−12.31 to −8.97]), **p < 0.05**	I^2^ = 94%, τ^2^ = 3.9, p < 0.01
	BoNT-A vs. baseline 52 weeks, CM	5/1579	MD: −10.32 (95% CI [−14.92 to −5.73]), **p < 0.05**	I^2^ = 99%, τ^2^ = 25, p < 0.01
Lindsay, 2024 [35]	BoNT-A vs. baseline, after 2−2.6 injection cycles (appr 6 months), CM	5/204	Hedge’s *g*: −0.97 (95% CI [−0.58 to −1.35]), **p < 0.001** Large effect size	I^2^ = 66%, τ^2^ = 0.1, p = 0.02
Chen, 2021 [31]	BoNT-A vs. placebo, 12 weeks, CM	NR/835	SMD: −0.58 (95% CI [−0.86 to −0.29]), **p < 0.05**	I^2^ = 83%, Q = 23, p < 0.001
	BoNT-A vs. galcanezumab 120 mg, 12 weeks, CM	6/2809	SMD: 0.31 (95% CI [−0.31 to 0.94]), NS	
	BoNT-A vs. fremanezumab 675 mg, 12 weeks, CM		SMD: 0.23 (95% CI [−0.39 to 0.86]), NS	
	BoNT-A vs. eptinezumab 300 mg, 12 weeks, CM		SMD: 0.17 (95% CI [−0.49 to 0.83]), NS	
	NMA, 12 weeks, CM	6/2809	BoNT-A: P score = 0.80	
			Eptinezumab 300 mg: P score = 0.62	
			Fremanezumab 675 mg: P-score = 0.53	
			Galcanezumab 120 mg: P score = 0.46	
Zheng, 2020 [29]	BoNT-A vs. placebo, 12 weeks, CM	9/948	SMD: −0.79 (95% CI [−1.15 to −0.43]), **p < 0.05**	I^2^ = 86%
	Topiramate vs. BoNT-A, 12 weeks, CM	NR	SMD: −0.27 (95% CI [−0.79 to 0.25]), NS	
	Acupuncture vs. BoNT-A, 12 weeks, CM	NR	SMD: −0.82 (95% CI [−1.48 to −0.16]) **p < 0.05**)	
	NMA, 12 weeks, CM	18/1203	Acupuncture: P score = 0.98	
			Topiramate: P score = 0.72	
			BoNT-A: P score = 0.53	
***Change in monthly migraine episodes***				
Shuhendler, 2009 [26]	Change from baseline BoNT-A vs. placebo, 90 days, EM	9/2090	SMD: −0.05 (95% CI [−0.13 to 0.04]), p = 0.28	I^2^ = 0%, chi^2^ = 16, p = 0.28
Herd, 2018 [7]	BoNT-A vs. placebo, 12 weeks, EM	3/1096	MD: 0.13 (95% CI [−0.17 to 0.43]), p = 0.41	I^2^ = 0%, Chi^2^= 1.5, p = 0.48
	BoNT-A vs. placebo, 12 weeks, CM	1/679	MD: 0.10 (95% CI [−0.71 to 0.91]), p = 0.30	NA
***Change in monthly headache episodes***				
Jackson, 2012 [27]	BoNT-A vs. placebo, 84 to 270 days, EM	9/1838	MD: 0.05 (95% CI [−0.26 to 0.36]), NS	I^2^ = 30%, Q = 11, p = 0.18
	BoNT-A vs. placebo, 84 to 270 days, CM	5/1508	MD: −2.30 (95% CI [−3.66 to −0.94]), **p < 0.05**	I^2^ = 32%, Q = 5.9, p = 0.21
Shen, 2020 [14]	BoNT-A vs. placebo, 84–395 days, EM	5/2770	MD: −0.01 (95% CI [−0.19 to 0.18]), NS	I^2^ = 0%, τ^2^ = 0, p = 0.77
	BoNT-A vs. placebo, 84–395 days, CM	6/241	MD: −1.68 (95% CI [−3.31 to −0.06]), **p < 0.05**	I^2^ = 61%, τ^2^ = 2.2, p = 0.02
Bruloy, 2018 [13]	BoNT-A vs. placebo, 3 months, EM	11/2837	MD: −0.17 (95% CI, [−0.41 to 0.08]), p = 0.18	I^2^ = 52%, chi2 = 52, p = 0.001
	BoNT-A vs. placebo, 3 months, CM	6/1546	MD: −1.56 (95% CI, [−3.05 to −0.07]), **p = 0.04**	I^2^ = 37%, chi^2^ = 7.9, p = 0.16
***≥50% reduction in monthly migraine frequency (days/episodes)***				
Lanteri-Minet, 2022 [33]	BoNT-A vs. baseline, 24 weeks, CM	7/1359	RR: 46.57 (95% CI [29.50 to 63.65]), **p < 0.05**	I^2^ = 95%, τ^2^ = 496, p < 0.01
Frank, 2021 [32]	BoNT-A vs. placebo, 12–38 weeks, EM	4/1047	OR: 1.14 (95% CI [0.76 to 1.70]), NS	NR
	BoNT-A vs. placebo, 12–38 weeks, CM	2/1425	OR: 1.51 (95% CI [1.10 to 2.09]), **p < 0.05**	NR
Shamliyan, 2013 [28]	BoNT-A vs. placebo, 6 months, CM	3/459	RR: 1.5 (95% CI [1.2 to 1.8]), **p < 0.05** NNTB: 6 (95% CI [4 to 12]	NR
Zhao et al., 2024 [10]	BoNT-A vs. placebo, time NR, CM	NR	RR: 0.96 (95% CI [0.55 to 1.69]), NS	NR
	Topiramate vs. BoNT-A, time NR, CM	NR	RR: 48.01 (95% CI [2.88 to 799.5]), **p < 0.05**	
	NMA, time NR, CM	9/4558	Topiramate: SUCRA = 0.99	
			Fremanezumab: SUCRA = 0.78	
			Galcanezumab: SUCRA = 0.66	
			Eptinezumab: SUCRA = 0.40	
			BoNT-A: SUCRA = 0.15	
***≥50% reduction in monthly headache frequency (days/episodes)***				
Jackson, 2012 [27]	BoNT-A vs. placebo, 84–270 days, EM	2/421	RR: 1.00 (95% CI [0.85 to 1.18]), NS	
	BoNT-A vs. placebo, 84–270 days, CM	2/92	RR: 2.21 (95% CI [1.30 to 3.78]), **p < 0.05**	I^2^ = 0%, Q = 0.1, p = 0.86)
Shen, 2020 [14]	BoNT-A vs. placebo, 84–395 days, EM	2/1100	RR: 1.13 (95% CI [0.92 to 1.38]), NS	I^2^ = 34%, τ^2^ = 0.1, p = 0.17
	BoNT-A vs. placebo, 84–395 days, CM	3/1476	RR: 1.42 (95% CI [0.61 to 3.31]), NS	I^2^ = 85%, τ^2^ = 0.4, **p < 0.01**
Chen, 2021 [31]	BoNT-A vs. placebo, 3 months, CM	NR/117	RR: 1.43 (95% CI [0.56 to 3.64)], NS	I^2^ = 79%, Q = 4.8, p = 0.028
	BoNT-A vs. galcanezumab 120 mg, 3 months, CM	NR	RR: 1.25 (95% CI [0.27 to 5.79]), NS	
	BoNT-A vs. eptinezumab 300 mg, 12 weeks, CM	NR	RR: 0.93 (95% CI [0.20 to 4.26]), NS	
	NMA, 12 weeks, CM	4/1826	Galcanezumab 120 mg: P score = 0.67	
			BoNT-A: P score = 0.57	
			Eptinezumab 300 mg: P score 0.53	

BoNT-A: botulinum toxin type-A; Chi^2^: Chi-square test; CI: Confidence interval; CM: Chronic migraine; EM: Episodic migraine; I^2^: Higgins test; MD: mean difference; NA: Not applicable; NMA: Network meta-analysis; NNTB: The number needed to treat to benefit; NR: Not reported; NS: Not significant; OR: Odds ration; p: p-value; Q: Cochrane’s Q; RR: Risk ratio; SMD: Standard mean difference; SUCRA: Surface under the cumulative ranking curve (reported as absolute numbers (0–1) instead of percent); τ^2^: Tau-squared; WMD: Weighted mean difference. Significant differences in outcomes are shown in bold font. Higher P scores and SUCRA indicate better effect.

**Table 2:** Effect of BoNT-A treatment on change in migraine severity.

Author, Year	Outcome Intervention(s)	Number of Studies/Participants	Results/Findings	Heterogeneity
Affatato, 2021 [30]	BoNT-A vs. baseline, 6 months, CM	2/75	MD: −1.70 (95% CI [−3.27 to −0.13]), **p = 0.03**	I^2^ = 99%, Q = 211, p = 0.00
Lindsay, 2024 [35]	BoNT-A vs. baseline, after 2–2.6 injection cycles (appr 6 months), CM	5/204	Hedge’s g: −1.24 (95% CI [−0.55 to −1.94]), **p = 0.0005**	I^2^ = 89, τ^2^ = 0.6, p = 0.00
Herd, 2018 [7]	BoNT-A vs. placebo 12 weeks, EM	1/32	MD: −4.90 (95% CI [−6.56 to −3.24]), **p < 0.001**	NA
	BoNT-A vs. placebo, 12 weeks, CM	2/75	MD: −2.70 (95% CI [−3.31 to −2.09]), **p < 0.001**	I^2^ = 0%, Chi^2^ = 0.0, p = 0.98
Shen, 2020 [14]	VAS, BoNT-A vs. placebo, 84–395 days, EM and CM combined	3/185	MD: −3.13 (95% CI [−4.82 to −1.43]), **p < 0.05**	I^2^ = 87%, τ^2^ = 2.6, p < 0.01

BoNT-A: botulinum toxin type-A; Chi^2^: Chi-square test; CI: Confidence interval; CM: Chronic migraine; EM: Episodic migraine; I^2^: Higgins test; MD: mean difference; NR: Not reported; p: p-value; Q: Cochrane’s Q; SMD: Standard mean difference; τ^2:^ Tau-squared; VAS: Visual analog scale. Significant differences in outcomes are shown in bold font.

**Table 3:** Effect of BoNT-A treatment on quality-of-life outcomes.

Author, Year	Outcome Intervention(s)	Number of Studies/Participants	Results/Findings	Heterogeneity
*HIT-6 score*				
Affatato, 2021 [30]	BoNT-A vs. baseline, 6 months, CM	3/102	MD: −5.60 (95% CI [−9.56 to 1.63]), **p = 0.01**	I^2^ = 99%; Q = 398, p = 0.00
Lanteri-Minet, 2022 [33]	BoNT-A vs. baseline, 24 weeks, CM	1/211	MD: –11.70 (95% CI [−13.86 to −9.54]), **p < 0.05**	NA
	BoNT-A vs. baseline, 52 weeks, CM	1/211	MD: –11.80 (95% CI [−14.70 to −8.90]), **p < 0.05**	NA
Shen, 2020 [14]	BoNT-A vs. placebo, 84–395 days, migraine	2/1452	MD: −4.03 (95% CI [−8.32 to 0.26]), NS	I^2^ = 57%, τ^2^ = 5.8, p = 0.13
Chen, 2021 [31]	BoNT-A vs. placebo, 12 weeks, CM	NR/117	SMD: −0.44 (95% CI [−1.35 to 0.48]), NS	I^2^ 88%; Q = 8.6, p = 0.003
	Fremanezumab 675 mg vs. BoNT-A, 12 weeks, CM	NR	SMD: –3.82 (95% CI [−5.37 to −2.28]), **p < 0.05**	
	Eptinezumab 300 mg vs. BoNT-A, 12 weeks, CM	NR	RR: −0.70 (95% CI [−2.25 to 0.85]), NS	
	NMA, 12 weeks, CM	4/1981	Fremanezumab 675 mg: P score = 0.88	
			Eptinezumab 300 mg: P score = 0.65	
			BoNT-A: P score 0.40	
***MIDAS score***				
Affatato, 2021 [30]	BoNT-A vs. baseline, 6 months, CM	3/92	MD: −36.86 (95% CI [−53.34 to −20.38]), **p < 0.01**	I^2^ 97%, Q = 56, p = 0.00
Lanteri-Minet, 2022 [33]	BoNT-A vs. baseline, 24 weeks, CM	4/425	MD: 44.74 (95% CI [28.50 to 60.99]), **p < 0.05**	I^2^ = 56%, τ ^2^ = 148, p = 0.08
Shen, 2020 [14]	BoNT-A vs. placebo, 84–395 days, migraine	1/68	MD: −15.80 (95% CI [−25.47 to −6.13]), **p < 0.05**	NA
Chen, 2021 [31]	BoNT-A vs. placebo, 12 weeks, CM	NR/117	SMD: −0.46 (95% CI [−0.72, −0.20]), **p < 0.05**	I^2^ = 0%, Q = 0.9, p = 0.339
	Galcanezumab 120 mg vs. BoNT-A, 12 weeks, CM	NR	SMD: −1.88 (95% CI [−2.20 to −1.56]), **p < 0.05**	
	NMA, 12 weeks, CM	3/1320	Galcanezumab 120 mg: P score = 1.00	
			BoNT-A: P-score = 0.33	
Zhao, 2024 [10]	BoNT-A vs. placebo, time NR, CM	NR	WMD: −7.55 (95% CI [−26.22 to 15.37]), NS	NR
	Topiramate vs. BoNT-A, time NR, CM	NR	WMD: −2.56 (95% CI [−30.43 to 35.41]), NS	
	Galcanezumab vs. BoNT-A, time NR, CM	NR	WMD: −0.51 95% CI [−29.41 to 33]), NS	
	NMA, time NR, CM	9/3410	Galcanezumab: SUCRA = 0.61	
			BoNT-A: SUCRA = 0.60	
			Topiramate: SUCRA = 0.56	
Zheng, 2020 [29]	BoNT-A vs. placebo, 12 weeks, CM	5/196	SMD: −0.71 (95% CI [−1.58 to 0.16]), NS	I^2^ = 93%
	Topiramate vs. BoNT-A, 12 weeks, CM	NR	SMD: 0.23 (95% CI [−0.73 to 1.19]), NS	
	Acupuncture vs. BoNT-A, 12 weeks, CM	NR	SMD: −1.01 (95% CI [−2.99 to 0.96]), NS	
	NMA, 12 weeks, CM	11/507	Acupuncture: P score = 0.91	
			BoNT-A: P score = 0.59	
			Topiramate: P score = 0.41	
***BDI and PHQ scores***				
Affatato, 2021 [30]	***BDI*** BoNT-A vs. baseline, 3 months, CM	2/87	MD: −8.94 (95% CI [−10.04 to −7.84]), **p < 0.01**	I^2^ = 79%, Q = 4.8, p = 0.03
	***BDI-II*** BoNT-A vs. baseline, 6 months, CM	2/84	MD: −5.90 (95% CI [−9.92 to −1.88]), **p < 0.01**	I^2^ = 97%, Q = 33, p = 0.00
	***PHQ-9*** BoNT-A vs. baseline, 6 months, CM	3/445	MD: −4.49 (95% CI [−4.58 to −4.39]), **p < 0.01**	I^2^ = 11%, Q = 1.1, p = 0.29
***MSQ and combined measures***				
Lanteri-Minet, 2022 [33]	***MSQ*** BoNT-A vs. baseline, 24 weeks, CM	1/972	MD: 23.60 (95% CI [21.56 to 25.64]), **p < 0.05**	NA
	***MSQ*** BoNT-A vs. baseline, 52 weeks, CM	1/972	MD: 30.90 (95% CI [28.29 to 33.51]), **p < 0.05**	NA
Bruloy, 2018 [13]	***HIT-6, MIDAS, BDI combined,*** BoNT-A vs. placebo, 3 months, EM	2/278	MD: −0.41 (95% CI [−0.79 to −0.03]), **p < 0.04**	I^2^ = 57%, Chi^2^ = 11, p = 0.04
	***HIT-6, MIDAS, BDI combined,*** BoNT-A vs. placebo, 3 months, CM	3/1520	MD: −0.39 (95% CI [−0.51 to −0.28]), **p < 0.001**	I^2^ = 14%, Chi^2^ = 3.5, p = 0.32
	***HIT-6, MIDAS, BDI combined,*** BoNT-A vs. placebo, 3 months, EM and CM combined	5/1798	MD: −0.43 (95% CI [−0.59 to −0.27]), **p < 0.001**	I^2^ = 41%, Chi^2^ = 15, p = 0.09

BDI: Beck’s depression index; BoNT-A: botulinum toxin type-A; Chi^2^: Chi-square test; CI: Confidence interval; CM: Chronic migraine; EM: Episodic migraine; HIT-6: Headache impact test; I^2^: Higgins test; MIDAS: Migraine disability assessment; MD: mean difference; MSQ: Migraine-specific quality of life questionnaire; NA: Not applicable; NMA: Network meta-analysis; NR: Not reported; NS: Not significant; p: p-value; PHQ-9: Patient history questionnaire-9; Q: Cochrane’s Q; RR: Risk ratio; SMD: Standard mean difference; SUCRA: Surface under the cumulative ranking curve (reported as absolute numbers (0–1) instead of percent); τ^2^: Tau-squared; WMD: Weighted mean difference. Significant differences in outcomes are shown in bold font. Higher P scores and SUCRA indicate better effect.

**Table 4:** Effect of BoNT-A treatment on use of rescue medication and adverse events.

Author, Year	Outcome Intervention(s)	Number of Studies/Participants	Results/Findings	Heterogeneity
*Use of rescue medicine*				
Lanteri-Minet, 2022 [33]	BoNT-A vs. baseline, 24 weeks,	3/378	MD: −7.40 (95% CI [−13.04 to −1.77]), **p < 0.05**	I^2^ = 85%, τ ^2^ = 18.9, p < 0.01
	BoNT-A vs. baseline, 52 weeks	3/378	MD: −5.99 (95% CI [−15.60 to 3.61]), NS	I^2^ = 99%, τ ^2^ = 66.4, p < 0.01
Herd, 2018 [7]	BoNT-A vs. placebo, 12 weeks	2/717	MD: −1.29 (95% CI [−3.09 to 0.52], *p* = 0.16)	I^2^ = 37%, Chi^2^ = 1.6, p = 0.16
Zheng, 2020 [29]	BoNT-A vs. placebo, 12 weeks	2/81	MD: −0.82 (95% CI [−1.39 to −0.25]), **p < 0.05**	I^2^ = 95%
	Topiramate vs. BoNT-A, 12 weeks	NR	MD: 0.67 (95% CI [0.07 to 1.27]), **p < 0.05**	
	Acupuncture vs. BoNT-A, 12 weeks	NR/168	MD: −0.23 (95% CI [−0.74 to 0.29]), NS	
	NMA, 12 weeks	7/386	Acupuncture: P score = 0.94	
			BoNT-A: P score = 0.73	
			Topiramate: P score = 0.26	
***All AEs***				
Bruloy, 2018 [13]	BoNT-A vs. placebo, 3 months	13/3146	RR: 1.32 (95% CI, 1.11 to 1.57), **p = 0.002**	I^2^ = 66%, Chi^2^ = 50.7, p < 0.0001
Jackson, 2012 [27]	BoNT-A vs. placebo, 84–270 days	25/2955	RR: 1.25 (95% CI [1.14 to 1.36]), **p < 0.05**	I^2^ = 61%, Q = 61, p = 0.15
Shamliyan, 2013 [28]	BoNT-A vs. placebo, 3–6 months	9/5031	RR: 1.6 (95% CI [1.3 to 2.0]), **p < 0.05** NNTH: 6 (95% CI [5 to 11])	I^2^ = 81%
Herd, 2018 [7]	BoNT-A vs. placebo, 12 weeks	13/3325	RR: 1.28 [95% CI [1.12 to 1.47]), **p = 0.0003**	I^2^ = 63%, Chi^2^ = 26.7, p = 0.003
	BoNT-A vs. topiramate and valproate,12 weeks	2/114	RR: 0.84 (95% CI [0.37 to 1.88]), NS	NR
Zhao, 2024 [10]	BoNT-A vs. placebo, time NR	NR	RR: 0.88, (95% CI [0.59 to 1.39], NS)	NR
	Topiramate vs. BoNT-A, time NR	NR	RR: 0.34, (95% CI [0.21 to 0.55]), **p < 0.05**	
	NMA, time NR	19/8067	Eptinezumab: SUCRA = 0.90	
			BoNT-A: SUCRA = 0.80	
			Galcanezumab: SUCRA = 0.49	
			Fremanezumab: SUCRA = 0.45	
			Topiramate: SUCRA = 0.08	
Zheng, 2020 [29]	BoNT- vs. placebo, 12 weeks	7/240	RR: 1.11 [0.74 to 1.66]), NS	NR
	NMA, 12 weeks	17/650	Topiramate: P score = 0.94	
			BoNT-A: P score = 0.62	
			Acupuncture: P score = 0.00	
	BoNT-A vs. placebo, 24 weeks	4/751 1/30	RR: 1.20 [1.11 to 1.31]), **p < 0.05**	
	NMA, 24 weeks	5/751	Topiramate: P score = 0.96	
			BoNT-A: P score = 0.54	
***Treatment-related AEs***				
Shen, 2020 [14]	BoNT-A vs. placebo, 84–395 days	16/3715	RR: 1.54 (95% CI [1.25 to 1.93]), **p < 0.05**	I^2^ = 80, p < 0.01
Corasaniti, 2023 [34]	BoNT-A vs. placebo, 3–56 months	4/3169	RR: 2.29 (95% CI [1.98 to 2.66]), **p < 0.001**	I^2^ = 0%, Chi^2^ = 2.19, p = 0.53
	BoNT-A vs. topiramate, 3–56 months	3/396	RR: 0.73 (95% CI [0.23 to 2.34]), p = 0.59	I^2^ = 96%, Chi^2^ = 46.4, p < 0.0001
Chen, 2021 [31]	BoNT-A vs. placebo, 12 weeks	NR/137	RR: 1.03 (95% CI [0.72 to 1.49]), NS	I^2^ = 0%; Q = 0.87, p = 0.647
	BoNT-A vs. galcanezumab 120 mg, 12 weeks	NR	RR: 1.73 (95% CI [0.60 to 5.05]), NS	
	BoNT-A vs. fremanezumab 675 mg, 12 weeks	NR	RR: 1.13 (95% CI [0.76 to 1.68]), NS	
	NMA, 12 weeks	5/2516	BoNT-A: P-score = 0.72.	
			Fremanezumab 675 mg: P score = 0.51	
			Galcanezumab 120 mg: P score = 0.24	
	BoNT-A vs. placebo, 24 weeks	NR/347	RR: 2.44 (95% CI [1.81 to 3.30]), **p < 0.05**	NA (too few studies)
	NMA, 24 weeks	2/1321	Eptinezumab 300 mg: P score = 0.52	
			BoNT-A: P-score = 0.02	
Zheng, 2020 [29]	BoNT-A vs. placebo, 12 weeks	3/137	RR: 1.03 [0.72 to 1.49]), NS	I^2^ = 0%
	Topiramate vs. BoNT-A, 12 weeks	NR	RR 1.46, (95% CI [0.96 to 2.22] NS	
	NMA, 12 weeks	4/290	Topiramate: P score = 0.96	
			BoNT-A: P score = 0.30	
	BoNT-A vs. placebo, 24 weeks	3/718	RR: 2.33 [1.86 to 2.92]), **p < 0.05**	
	Topiramate vs. BoNT-A, 24 weeks	NR	RR 1.39 (95% CI [1 to 1.94]), NS	
	NMA, 24 weeks	4/748	Topiramate: P score = 0.99	
			BoNT-A: P score = 0.51	

AE: Adverse event; BoNT-A: botulinum toxin type-A; Chi^2^: Chi-square test; CI: Confidence interval; I^2^: Higgins test; MD: mean difference; NMA: Network meta-analysis; NA: Not applicable; NNTH: The number needed to treat to harm; NR: Not reported; NS: Not significant; p: p-value; Q: Cochrane’s Q; RR: risk ratio; SUCRA: Surface under the cumulative ranking curve (reported as absolute numbers (0–1) instead of percent). Significant differences in outcomes are shown in bold font. Higher P scores and SUCRA indicate less AEs, with the exception of in Zheng et al. [29], where higher P scores indicate more AEs.

**Table 5:** Evidence from quantitative research synthesis regarding the effect of BoNT-A treatment on headache frequency and severity, as well as adverse events.

**Author, Year**	**Diagnosis**	**Headache Frequency as Primary Outcomes**
*BoNT-A* **vs.** *baseline or placebo*		
Bruloy, 2018 [13]	EM	Non-significant improvement with negligible effect compared to placebo for number of headache episodes
Herd, 2018 [7]	EM	Non-significant improvement with negligible effect compared to placebo number of migraine days
Frank, 2021 [32]	EM	Non-significant improvement with negligible effect compared to placebo ≥ 50% reduction in migraine days
Jackson, 2012 [27]	EM	Negligible effect compared to placebo for number of headache episodes
Shen, 2020 [14]	EM	Negligible effect compared to placebo for number of headache episodes
Shuhendler, 2009 [26]	EM	Non-significant improvement with negligible effect compared to placebo for number of migraine episodes
Affatato, 2021 [30]	CM	Significant improvement after BoNT-A compared to baseline for number of migraine days
Lanteri-Minet, 2022 [33]	CM	Significant improvement after BoNT-A compared to baseline for number of headache days
Lindsay, 2024 [35]	CM	Significant improvement after BoNT-A compared to baseline for number of headache episodes
Bruloy, 2018 [13]	CM	Effect favoring BoNT-A compared to placebo for number of headache episodes
Chen, 2021 [31]	CM	Significant improvement compared to placebo for number of headache days, but non-significant effect for number of migraine days
Frank, 2021 [32]	CM	Effect favoring BoNT-A compared to placebo for ≥50% reduction in migraine days
Herd, 2018 [7]	CM	Effect favoring BoNT-A compared to placebo for number of migraine days
Jackson, 2012 [27]	CM	Significant improvement after BoNT-A compared to placebo for number of headache episodes
Shamliyan, 2013 [28]	CM	Effect favoring BoNT-A compared to placebo for ≥50% reduction in migraine episodes
Shen, 2020, [14]	CM	Effect favoring BoNT-A compared to placebo for the number of headache episodes, but non-significant effect for ≥50% reduction in headache episodes
Zhao, 2024 [10]	CM	Effect favoring BoNT-A compared to placebo for number of headache days, but non-significant effect for migraine days
Zheng, 2020 [29]	CM	Effect favoring BoNT-A compared to placebo for number of headache days, but non-significant effect for number of migraine days
***BoNT-A*** **vs.** ***other treatments***		
Chen, 2021 [31]	CM	Non-significant effect compared to eptinezumab, fremanezumab, and galcanezumab for headache days
Chen, 2021 [31]	CM	Effect favoring galcanezumab and fremanezumab compared to BoNT-A migraine days
Zhao, 2024 [10]	CM	Non-significant effect compared to topiramate, propranolol, eptinezumab, erenumab, valproate, fremanezumab, flunarizine, and galcanezumab for number of migraine days. For ≥50% reduction in days, topiramate and fremanezumab were the most effective
Zheng, 2020 [29]	CM	Effect favoring topiramate compared to BoNT-A for number of migraine days. Effect favoring topiramate and acupuncture for number of headache days
**Author, year**	**Diagnosis**	**Headache frequency as secondary outcomes**
Jackson, 2012 [27]	EM	Negligible effect compared to placebo for > 50% red headache ep
Herd, 2018 [7]	EM	Non-significant improvement with negligible effect compared to placebo for migraine ep
Chen, 2021 [31]	CM	Non-significant improvement with negligible effect compared to placebo for ≥50% red headache ep
Herd, 2018 [7]	CM	Significant improvement compared to placebo for headache days, but non-significant effect for number of migraine episodes
Jackson, 2012 [27]	CM	Effect favoring BoNT-A compared to baseline for ≥50% reduction in headache episodes
Lanteri-Minet, 2022 [33]	CM	Significant improvement after BoNT-A compared to baseline for ≥50% reduction in migraine days
Zhao, 2024 [10]	CM	Non-significant improvement with negligible effect compared to placebo for ≥50% reduction in migraine days
Zheng, 2020 [29]	CM	Non-significant improvement with negligible effect compared to placebo for ≥50% red headache days
**Author, year**	**Diagnosis**	**Migraine severity**
***BoNT-A*** **vs.** ***baseline or placebo***		
Affato, 2021 [30]	CM	Significant improvement after BoNT-A compared to baseline
Lindsay, 2024 [35]	CM	Significant improvement after BoNT-A compared to baseline
Herd, 2018 [7]	CM and EM	Significant improvement after BoNT-A compared to placebo
Shen, 2020 [14]	CM and EM	Significant improvement after BoNT-A compared to placebo
**Author, year**	**Diagnosis**	**Adverse events**
***BoNT-A*** **vs.** ***baseline or placebo***		
Bruloy, 2018 [13]	NA	Indicates moderate risk for adverse events
Chen, 2021 [31]	NA	Indicates high risk for adverse events
Corasaniti, 2023 [34]	NA	Indicates high risk for adverse events
Herd, 2018 [7]	NA	Indicates moderate risk for adverse events
Jackson, 2012 [27]	NA	Indicates moderate risk for adverse events
Shamliyan, 2013 [28]	NA	Indicates high risk for adverse events and high risk of discontinuation
Zhao, 2024 [10]	NA	Indicates low risk for adverse events
Zheng, 2020 [29]	NA	Indicates high risk for adverse events
***BoNT-A*** **vs.** ***other treatments***		
Chen, 2021 [31]	NA	No significant differences to galcanezumab and fremanezumab, but high risk
Corasaniti, 2023 [34]	NA	No significant differences to topiramate, but high risk
Herd, 2018 [7]	NA	Favors BoNT-A compared to topiramate and valproate, but moderate risk
Zhao, 2024 [10]	NA	Significantly fewer adverse events than topiramate, but more than erenumab; however, still low risk
Zheng, 2020 [29]	NA	Favors BoNT-A compared to topiramate (even though not significant), but high risk

BoNT-A: Botulinum toxin type-A; CM: Chronic migraine; EM: Episodic migraine. Green color indicates a positive outcome in the synthesis. For treatment outcomes, this indicates an effect favoring BoNT-A in comparison to either placebo or baseline, or other treatments of CM and EM (chronic and episodic migraine). For adverse events, the green color indicates low risk for adverse events. Orange color indicates an uncertain outcome in the synthesis. For treatment outcomes, this indicates an effect that does not favor BoNT-A but can be similar to either placebo or other treatments of CM and EM. For adverse events, the orange color indicates moderate risk for adverse events. Red color indicates an absence of, or very low, effect outcomes in the synthesis. For treatment outcomes, this indicates an effect that does not favor BoNT-A but instead favors either placebo or other treatments of CM and EM. For adverse events, the red color indicates a high risk for adverse events.

## Data Availability

No new data were created or analyzed in this study.

## Supplementary Materials

The following supporting information can be downloaded at: https://www.mdpi.com/article/10.3390/toxins18010033/s1, Table S1: Quality assessment of the studies according to the Umbrella Review methodology working group [53]; Table S2: Citations not deemed as high quality in quality assessment according to the Umbrella Review methodology working group. Table S3: Description of included studies; Table S4: Primary articles from the 14 meta-analyses included in the umbrella review; Table S5: Preferred Reporting Items for Systematic reviews and Meta-Analyses (PRISMA) Checklist. Table S6: Search strategies for each database: (1) Medline; (2) Embase; (3) Cochrane; (4) Web of Science Core Collection; (5) Cinahl.

## Abbreviations

The following abbreviations are used in this manuscript:

BDI	Becks Depression Index
BoNT-A	Botulinum toxin type-A
CGRP	Calcitonin gene-related peptide
CGRPmAbs	CGRP monoclonal antibodies
HIT-6	Headache Impact Test-6
MeSH	Medical Subject Headings
MIDAS	Migraine Disability Assessment
MSQ	Migraine-Specific Questionnaire
PHQ-9	Patient History Questionnaire-9
PICOTS	Population, Intervention, Comparators, Outcomes, Timing, and Study design
PREEMT	Phase III Research Evaluating Migraine Prophylaxis Therapy
PRISMA	Preferred Reporting Items for Systematic Reviews and Meta-Analyses
QoL	Quality of life
SP	Substance P
SRs	Systematic reviews
VAS	Visual Analog Scale
