# Supplementary material for: Botulinum Toxin Type A for the Prevention of Migraines: An Umbrella Review of Systematic Reviews
Source: Toxins (Basel). 2026 Jan 9;18(1):33. doi: 10.3390/toxins18010033 (PMC12846396; doi:10.3390/toxins18010033)
Supplement: Supplementary file 1 [file toxins-18-00033-s001.zip › toxins-4077732-supplementary.pdf]

Supplemental materials: Botulinum Toxin Type A for the Prevention of Migraines: An Umbrella Review of Systematic Reviews

Goli Chamani, Hajer Jasim, Ava Minston, Marlon Ferreira Diaz, Rodrigo Lorenzi Poluha, Daniela Godoi Gonçalves, Maria Christidis, Essam Ahmed Al-Moraissi, Nikolaos Christidis, Giancarlo De la Torre Canales, Malin Ernberg

Table S1. Quality assessment of the studies according to the Umbrella Review methodology working group [50].

Author, year	Q1.	Q2.	Q3.	Q4.	Q5.	Q6.	Q7.	Q8.	Q9.	Q10.	Methodological quality / risk of bias
Affatato et al, 2021	Meet	Meet	Meet	Meet	Meet	Meet	Meet	Meet	Meet	Meet	High quality/low risk of bias
Argyriou et al, 2021	Meet	Meet	Meet	Meet	Meet	Meet	N/A	Not meet	Meet	Meet	Low quality/high risk of bias
Azadvari et al, 2023	Meet	Meet	Meet	Meet	Meet	Unclear	Meet	Meet	Meet	N/A	Some concerns
Blumenfeld et al, 2023	Meet	Meet	Meet	Meet	Meet	Unclear	N/A	Not meet	Meet	Meet	Low quality/high risk of bias
Bruloy et al, 2018	Meet	Meet	Meet	Meet	Meet	Meet	Meet	Meet	Meet	Meet	High quality/low risk of bias
Chen et al, 2021	Meet	Meet	Meet	Meet	Meet	Meet	Meet	Meet	Meet	Meet	High quality/low risk of bias
Cho et al, 2017	Meet	Meet	Meet	Meet	Meet	Meet	N/A	Not meet	Meet	Meet	Low quality/high risk of bias
Corasaniti et al, 2023	Meet	Meet	Meet	Meet	Meet	Meet	Meet	Meet	Meet	Meet	High quality/low risk of bias
Frank et al, 2021	Meet	Meet	Meet	Meet	Meet	Meet	Meet	Meet	Meet	Meet	High quality/low risk of bias
Gómez-Dabó et al, 2024	Meet	Meet	Meet	Meet	Meet	Meet	N/A	Not meet	Meet	Meet	Low quality/high risk of bias
Gottschalk et al, 2022	Meet	Meet	Meet	Meet	Meet	Meet	N/A	Not meet	Meet	Meet	Low quality/high risk of bias
Herd et al, 2018	Meet	Meet	Meet	Meet	Meet	Meet	Meet	Meet	Meet	Meet	High quality/low risk of bias

**Herd et al, 2019**	Meet	Meet	Meet	Meet	Meet	Meet	Meet	Not meet	Meet	Meet	Low quality/high risk of bias
**Jackson et al, 2012**	Meet	Meet	Meet	Meet	Meet	Meet	Meet	Meet	Meet	Meet	High quality/low risk of bias
**Khanal et al, 2022**	Meet	Meet	Meet	Meet	Meet	Meet	N/A	Not meet	Meet	Meet	Low quality/high risk of bias
**Kumar, 2006**	Meet	Meet	Meet	Meet	Meet	N/A	Not meet	Not meet	Meet	Meet	Low quality/high risk of bias
**Lanteri-Minet et al, 2022**	Meet	Meet	Meet	Meet	Meet	Meet	Meet	Meet	Meet	Meet	High quality/low risk of bias
**Lindsay et al, 2024**	Meet	Meet	Meet	Meet	Meet	Meet	Meet	Meet	Meet	Meet	High quality/low risk of bias
**Marcelo et al, 2020**	Meet	Meet	Meet	Meet	Meet	Meet	Meet	Not meet	Meet	Meet	Low quality/high risk of bias
**Mavridi et al, 2024**	Meet	Meet	Meet	Meet	Unclear	Meet	N/A	Not meet	Meet	Meet	Low quality/high risk of bias
**Mistry et al, 2023**	Meet	Meet	Unclear	Unclear	Unclear	Unclear	Meet	Not meet	Meet	Meet	Low quality/high risk of bias
**Naghdin et al, 2023**	Meet	Meet	Meet	Meet	Meet	Meet	Meet	Not meet	Meet	Meet	Low quality/high risk of bias
**Nauman n et al, 2008**	Meet	Meet	Meet	Meet	Meet	Unclear	N/A	Not meet	Meet	Meet	Low quality/high risk of bias
**Numtha vaj et al, 2024**	Meet	Meet	Meet	Meet	Meet	Meet	Meet	Not meet	N/A	Meet	Not a SR, just a protocol
**Oskoui et al, 2019**	Meet	Meet	Meet	Meet	Meet	Meet	Meet	Not meet	Meet	Meet	Low quality/high risk of bias
**Shamliy an et al, 2013**	Meet	Meet	Meet	Meet	Meet	Meet	Meet	Meet	Meet	Meet	High quality/low risk of bias
**Shateria n et al, 2022**	Meet	Meet	Meet	Meet	Unclear	Meet	N/A	Not meet	Meet	Meet	Low quality/high risk of bias
**Shen et al, 2020**	Meet	Meet	Meet	Meet	Meet	N/A	Meet	Meet	Meet	Unclear	High quality/low risk of bias
**Shuhend ler et al, 2009**	Meet	Meet	Meet	Meet	Meet	Meet	Meet	Meet	Meet	Meet	High quality/low risk of bias

**Swerts et al, 2021**	Meet	Meet	Meet	Meet	Meet	Meet	Meet	Not meet	Meet	Meet	Low quality/high risk of bias
**Tsou et al, 2021**	Meet	Meet	Meet	Meet	Meet	Unclear	Meet	Meet	Meet	Meet	Low quality/high risk of bias
**Webster et al, 2022**	Meet	Meet	Meet	Meet	Meet	Meet	Meet	Not meet	N/A	N/A	Not a SR, just a protocol
**Zheng et al, 2020**	Meet	Meet	Meet	Meet	Meet	Meet	Meet	Meet	Meet	Meet	High quality/low risk of bias
**Zhao et al, 2024**	Meet	Meet	Meet	Meet	Meet	Meet	Meet	Meet	Meet	Meet	High quality/low risk of bias

Q1: Is the review question clearly and explicity stated?
Q2: Were the inclusion criteria appropriate for the review question?
Q3: Was the search strategy appropriate?
Q4: Were the sources and resources used to search for studies adequate?
Q5: Were the criteria for appraising studies appropriate?
Q6: Was critical appraisal conducted by two or more reviewers independently?
Q7: Were the methods used to combine studies appropriate?
Q8: Was the likelyhood of publication bias assessed?
Q9: Were recommendations for policy and/or practice supported by the reported data?
Q10. Were the specific directives for new research appropriate?
N/A: Not applicable. All of questions 1, 2, 3, 4, 5, 6, 8, and 9 must be "Meet" (green colour) to be deemed of high quality/low risk of bias and thus included in the review.

Table S2. Articles read in full but not deemed as high quality in quality assessment according to the Umbrella Review methodology working group [50] and therefore excluded.

	Citation
1	Argyriou AA, Mitsikostas DD, Mantovani E, Vikelis M, Tamburin S. Beyond chronic migraine: a systematic review and expert opinion on the off-label use of botulinum neurotoxin type-A in other primary headache disorders. Expert Rev Neurother. 2021 Aug;21(8):923-944. doi: 10.1080/14737175.2021.1958677.
2	Azadvari M, Hosseini M, Razavi SZ, Ghajarzadeh M, Vaheb S. Safety Profile of Botulinum Toxin for Migraine Headache Prophylaxis: A Systematic Review and Meta-analysis. Ann Mil Health Sci Res. 2023 September; 21(3):e13922. doi: 10.5812/amh-139223.
3	Blumenfeld AM, Kaur G, Mahajan A, Shukla H, Sommer K, Tung A, Knievel KL. Effectiveness and Safety of Chronic Migraine Preventive Treatments: A Systematic Literature Review. Pain Ther. 2023 Feb;12(1):251-274. doi: 10.1007/s40122-022-00452-3.
4	Cho SJ, Song TJ, Chu MK. Treatment Update of Chronic Migraine. Curr Pain Headache Rep. 2017 Jun;21(6):26. doi: 10.1007/s11916-017-0628-6.
5	Gómez-Dabó L, Caronna E, Mas-de-Les-Valls R, Gallardo VJ, Alpuente A, Torres-Ferrus M, Pozo-Rosich P. Effectiveness and Safety of OnabotulinumtoxinA in Adolescent Patients with Chronic Migraine. Toxins (Basel). 2024 May 11;16(5):221. doi: 10.3390/toxins16050221.
6	Gottschalk C, Buse DC, Marmura MJ, Torphy B, Pavlovic JM, Dumas PK, Lalvani N, Blumenfeld A. The importance of an early onset of migraine prevention: an evidence-based, hypothesis-driven scoping literature review. Ther Adv Neurol Disord. 2022 May 31;15:17562864221095902. doi: 10.1177/17562864221095902.
7	Gupta VK. Botulinum Toxin—A Treatment for Migraine? A Systematic Review. Pain Med. 2006 Sep-Oct;7(5):386-94. doi: 10.1111/j.1526-4637.2006.00216.x.
8	Herd CP, Tomlinson CL, Rick C, Scotton WJ, Edwards J, Ives NJ, Clarke CE, Sinclair AJ. Cochrane systematic review and meta-analysis of botulinum toxin for the prevention of migraine. BMJ Open. 2019 Jul 16;9(7):e027953. doi: 10.1136/bmjopen-2018-027953.
9	Khanal S, Underwood M, Naghdi S, Brown A, Duncan C, Matharu M, Mistry H. A systematic review of economic evaluations of pharmacological treatments for adults with chronic migraine. J Headache Pain. 2022 Sep 16;23(1):122. doi: 10.1186/s10194-022-01492-y.
10	Marcelo R, Freund B. The Efficacy of Botulinum Toxin in Pediatric Chronic Migraine: A Literature Review. J Child Neurol. 2020 Oct;35(12):844-851. doi: 10.1177/0883073820931256.
11	Mavridi A, Redmond A, Archontakis-Barakakis P, Bogdanova-Mihaylova P, Deligianni CI, Mitsikostas DD, Mavridis T. Onabotulinumtoxina in the Prevention of Migraine in Pediatric Population: A Systematic Review. Toxins (Basel). 2024 Jun 28;16(7):295. doi: 10.3390/toxins16070295.
12	Mistry H, Naghdi S, Underwood M, Duncan C, Madan J, Matharu M. Competing treatments for migraine: a headache for decision-makers. J Headache Pain. 2023 Dec 5;24(1):162. doi: 10.1186/s10194-023-01686-y.
13	Naghdi S, Underwood M, Madan J, Brown A, Duncan C, Matharu M, Aksentyte A, Davies N, Rees S, Cooklin A, Grove A, Mistry H. Clinical effectiveness of pharmacological interventions for managing chronic migraine in adults: a systematic review and network meta-analysis. J Headache Pain. 2023 Dec 6;24(1):164. doi: 10.1186/s10194-023-01696-w.
14	Naumann M, So Y, Argoff CE, Childers MK, Dykstra DD, Gronseth GS, Jabbari B, Kaufmann HC, Schurch B, Silberstein SD, Simpson DM; Therapeutics and Technology Assessment Subcommittee of the American Academy of Neurology. Assessment: Botulinum neurotoxin in the treatment of autonomic disorders and pain (an evidence-based review) [RETIRED]: report of the Therapeutics and Technology Assessment Subcommittee of the American Academy of Neurology. Neurology. 2008 May 6;70(19):1707-14. doi: 10.1212/01.wnl.0000311390.87642.d8.
15	Oskoui M, Pringsheim T, Billinghurst L, Potrebic S, Gersz EM, Gloss D, Holler-Managan Y, Leininger E, Licking N, Mack K, Powers SW, Sowell M, Victorio MC, Yonker M, Zanitsch H, Hershey AD. Practice

guideline update summary: Pharmacologic treatment for pediatric migraine prevention: Report of the Guideline Development, Dissemination, and Implementation Subcommittee of the American Academy of Neurology and the American Headache Society. Neurology. 2019 Sep 10;93(11):500-509. doi: 10.1212/WNL.0000000000008105.

16 Shaterian N, Shaterian N, Ghanaatpisheh A, Abbasi F, Daniali S, Jahromi MJ, Sanie MS, Abdoli A. Botox (OnabotulinumtoxinA) for Treatment of Migraine Symptoms: A Systematic Review. Pain Res Manag. 2022 Mar 31;2022:3284446. doi: 10.1155/2022/3284446.

17 Swerts DB, Benedetti F, Peres MFP. Different routes of administration in chronic migraine prevention lead to different placebo responses: a meta-analysis. Pain. 2022 Mar 1;163(3):415-424. doi: 10.1097/j.pain.0000000000002365.

18 Tsou AY, Rouse B, Bloschichak A, Treadwell JR. Drugs and Devices for Migraine Prevention: Interactive Evidence Maps [Internet]Washington (DC): Patient-Centered Outcomes Research Institute (PCORI); 2021 Feb.

Table S3. Description of included studies.

Author, year<br><br>Country<br><br>Type of review	Objectives of included review	Intevention (I)<br><br>Control (C)	Participants details	Number of databases sourced and searched and date range of database searching	Number of studies, types of studies and country of origin of studies included in each review	Publication date range of studies included in the review that inform each outcome of interest	Instrument used to appraise the primary studies and the rating of their quality	Outcomes reported that are relevant to the umbrella review question	Method of synthesis/ analysis employed to synthesize the evidence
Affatato et al, 2021<br><br>Country Sweden (Italy, Russia, Turkey)<br><br>Type of review MA	Assess the effect of treatment with BONT-A on patients with chronic migraine and major depressive disorder compared to any of these disorders alone	I: After BoNT-A<br><br>C: Before BoNT-A<br><br>PREEMT Protocol	Disorder CM (IHS) and depression (BDI, BDI-II, PHQ-9)<br><br>N = 1492 F = 85.1%<br><br>Age range Adults (> 18 years)<br><br>Mean age range 34.7-54.1 years	Databases 3 (PubMed, Web of Science, Scopus)<br><br>Date range Until October 30, 2020	Number studies 8 studies (2 retrospective, 6 prospective)<br><br>Country of origin USA (2), Canada (1), Germany (1), Italy (2), Turkey (2)<br><br>5 studies CM<br>3 studies CM+depression	2015-2019	Primary studies<br><br>Qualitative analysis + data extraction table<br><br>Quality assessment<br>Not reported	Outcomes<br>1. Change from baseline of monthly migraine episodes<br>2. Change from baseline of HIT6<br>3. Change from baseline of MIDAS<br>4. Change from baseline of VAS<br>3. Change from baseline of depression (BDI, BDI-II, PHQ-9)<br><br>Time frame<br>3 months (BDI)<br>6 months (all other)	Data synthesis MD (95% C!)<br><br>Random-effects Model (Empirical Bayesian method) Forest plots<br><br>Heterogeneity Cochran's Q test and Higgins I^2 statistics<br><br>Publication bias Could not be assessed<br><br>$p < 0.05$
Bruloy et al, 2018<br><br>Country France<br><br>Type of review MA	Assess the effectiveness of BONT-A on changes in migraine frequency, impact	I: BoNT-A<br><br>C: Placebo<br><br>PREEMT Protocol	Disorder Migraine, (IHS), 57.5% EM, 42.5% CM<br><br>N = 3646 F = 86.2%	3 Databases (MEDLINE, EMBASE, Cochrane Library)<br><br>Date range Inception	17 studies (RCTs)<br><br>6 studies CM<br>11 studies EM	2000-2015	Primary studies: Data extraction table<br><br>Quality assessment Review	Outcomes<br>1. Change of monthly migraine episodes<br>2. Change of QoL (HIT-6, MIDAS, BDI combined)	Data synthesis MD (95% C!)<br>SMD (95% CI) for QoL<br><br>Random effects model

	on QoL, and safety versus placebo		Age range Adults (> 18 years)<br><br>Mean (range) age 42.8 years (18 to 65 years)	to August 2016	Country of origin Global (2), North America (8), Chile (1), Brazil (1), Germany (2), Italy (1) Thailand (1), China (1)		manager program	3. AE<br><br>Time frame 2 and 3 months	Forest plots<br><br>Heterogeneity Chi² test and Higgins I² statistics<br><br>Publication bias Funnel plots
Chen et al, 2021<br><br>Country China<br><br>Type of review NMA	To compare calcitonin gene-related peptide monoclonal antibodies with BONT-A in the preventive treatment of chronic migraine	I: BONT-A<br><br>C: Placebo	Disorder: CM (IHS) N = 4678 F = 82.0%<br><br>Age range Adults<br><br>Mean (range) age) 43.1 years (37.0 to 48.8) years	Databases 4 (OVID MELINE, EMBASE, Cochrane)<br><br>Date range Inception to September 2019	10 studies (RCTs) Country of origin USA (5), Brazil (1), UK (1), the Netherlands (1), Germany (1), Italy (1)<br><br>7 studies of BONT-A vs placebo	2004-2019	Primary studies: Qualitative analysis and Data extraction table<br><br>Quality assessment Cochrane risk of bias tool	Outcomes: 1. Change of montly migraine days 2. Change of monthly headache days 3. ≥50% reduction of monthly headache days 4. Change of HIT-6 5. Change of MIDAS 6. Adverse events<br><br>Time frame 4, 8, and 12 weeks	Data synthesis RR (95% C!) SMD (95% CI)<br><br>Bucher's method adjusted for variance using Rücker's method Forest plots<br><br>P score for treatment comparisons<br><br>Heterogeneity Cochran's Q test and Higgins I² statistics<br><br>Publication bias Sensitivity analysis
Corasaniti et al, 2023<br><br>Country Italy<br><br>Type of review MA	To critically appraise all the existing knowledge concerned with the safety	I: BONT-A<br><br>C1: Placebo<br><br>C2: Other active treatment	Disorder CM (IHS)<br><br>N = 3632 F = Not reported Age range	3 (MEDLINE, Scopus, Web of Science)<br><br>Date range	9 studies<br><br>7 in MA<br><br>Country of origin Not reported	2009-2020	Primary studies Qualitative analysis and Data extraction table	Outcomes 1. Adverse events<br><br>Time frame 3 to 56 months	Data synthesis RR (95% CI)<br><br>Random effect model Forest plots

	of BONT-A in CM	PREEMPT protocol	Adults (18-65 years)<br><br>Mean (range) age<br>Not reported	Until March 2, 2023			Quality assessment<br>the revised Cochrane RoB2 tool for RCTs		Heterogeneity<br>Chi² test and Higgins I² statistics<br><br>Publication bias<br>Egger's regression test
Frank et al, 2021<br><br>Country<br>Austria<br><br>Type of review<br>MA	To evaluate the efficacy as expressed with the 50% response rate for topiramate (TPM), BONT-A, and CGRP pathway monoclonal antibodies (CGRPmABs).	I: BONT-A<br><br>C: Placebo	Disorder<br>EM and CM<br><br>N = 2472<br>F = Not reported<br><br>Age range<br>Not reported<br><br>Mean (range) age<br>38.4 (21.6-46.2) years	3 (CENTRAL, Embase, MEDLINE)<br><br>Date range<br>Until March 20, 2020	32 studies RCTs<br><br>6 studies BONT-A<br><br>Country of origin<br>Not reported	2000-2011	Primary studies<br>Data extraction table<br><br>Quality assessment<br>Cochrane collaboration toll	Outcomes<br>1. ≥50% reduction of monthly migraine days (response rate)<br>2. Adverse events<br><br>Time frame<br>12-38 weeks	Data synthesis<br>OR (95% CI) 50% pain reduction<br><br>Random effect model<br>Forest plots<br><br>Heterogeneity<br>Higgins I² statistics<br><br>Publication bias<br>Egger's regression test and Funnel plots
Heard et al, 2018<br><br>Country<br>UK<br><br>Type of review<br>MA (Cochrane)	To assess the effects of BONT-A versus placebo or active treatment for the prevention or reduction in frequency of chronic or episodic migraine in adults	I: BONT-A<br><br>C1: Placebo<br><br>C2: Other prophylactics<br><br>C3: Different BONT-A doses	Disorder<br>CM and EM<br><br>N = 4190<br>F = 85%<br><br>Age range<br>Adults (> 18 years)<br><br>Mean (range) age<br>42 years	6 (CENTRAL, MEDLINE & MEDLINE in Process, EMBASE, ClinicalTrials.gov, World Health Organization International Clinical Trials Registry)<br><br>Date range	28 studies RCT<br><br>Country of origin<br>USA (16), Mexico (2), Chile (1), Brazil (2), Europe (1), Germany (2), Italy (1), India (1), Thailand (1), China (1)	2000-2016	Primary studies<br>Data extraction tables<br><br>Quality assessment<br>Cochrane Handbook for Systematic Reviews of Interventions	Outcomes<br>1. Change of monthly migraine days<br>2. Change of monthly migraine episodes<br>3. Chnge of headache intensity (VAS)<br>4. Use of rescue medication<br>5. Adverse events<br><br>Time frame	Data synthesis<br>MD (95%CI)<br>RR (95%CI)<br>NNTB (95%CI)<br>NNTH (95%CI)<br><br>Random effects model<br>Forest plots<br>Heterogeneity<br>Chi² test and

				Until December 2017	24 studies BONT-A vs placebo<br><br>3 studies BONT-A vs other prophylactics<br><br>21 studies in MA			12 weeks	Higgins I^2 statistics<br><br>Publication bias Funnel plots<br><br>Level of evidence GRADE $p < 0.05$
Jackson et al, 2012<br><br>Country USA (Japan)<br><br>Type of review MA	To assess BONT-A for the prophylactic treatment of headaches in adults.	I: BONT-A<br><br>C1: Placebo<br><br>C2: Other interventions	Disorder CM and EM (IHS)<br><br>N = 3713 F = 76%<br><br>Age range Adults (> 18 years)<br><br>Mean (range) age 42.1 years	3 (MEDLINE, EMBASE, Cochrane)<br><br>Date range 1966 to March 15, 2012	31 studies RCTs<br><br>17 BONT-A vs. placebo in migraine 4 BONT-A vs other prophylactics<br><br>Country of origin USA (4), North America (6), Brazil (1), Europe (1), Germany (2), Italy (1), India (1), Thailand (1)	2000-2011	Primary studies Data extraction tables<br><br>Quality assessment Cochrane Risk of Bias assessment tool, Jadad scale	Outcomes 1. Change of monthly headache episodes 2. ≥50% reduction of monthly headache episodes 3. Adverse events<br><br>Time frame 84 to 270 days	Data synthesis WMD (95% CI) SMD (95% CI)<br><br>Random-effects model (Higgins) Forest plots<br><br>Heterogeneity Cochran's Q and Higgins I^2 statistics<br><br>Publication bias Peters et al methods, Egger's regression test
Lanteri-Minet et al, 2022<br><br>Country France<br><br>Type of review MA	To conduct a metaanalysis to evaluate the real-world effectiveness of BONT-A for the	I: After BONT-A<br><br>C: Before BONT-A<br><br>PREEMPT protocol	Disorder CM (IHS)<br><br>N = 6562 F = 71% to 92.5%<br><br>Age range Adults (> 18 years)	3 (MEDLINE, EMBASE, Cochrane Library/Cochrane Central Register of Controlled Trials) Date range	42 studies (29 prospective, 13 retrospective)<br><br>Country of origin Global (1), Canada	2014-2020	Primary studies Data extraction tables<br><br>Quality assessment Only studies with ≥ 4 on the	Outcomes 1. Change from baseline of monthly headache days 2. ≥ 50% reduction from baseline of monthly	Data synthesis MD (95% CI)<br><br>Random-effects models Forest plot Heterogeneity

	treatment of chronic migraine		Mean (range) age<br>39.3 to 51.6 years	January 1, 2010 to March 31, 2021	(1), Italy (16), UK (3), Germany (1), France (1), Spain (11), Turkey (2), Taiwan (1), Korea (1), Australia (1), NR (3)		Newcastle–Ottawa Scale (NOS) were included	migraine days<br>3. Acute medication intake<br>4. Change from baseline of HIT-6<br>5. Change from baseline of MSQ<br>6. Change from baseline of MIDAS<br><br>Time frame<br>12 and 24 weeks	τ^2 test and Higgins I^2 statistics<br><br>Publication bias<br>Not reported
Lindsay et al, 2024<br><br>Country<br>Canada (USA)<br><br>Type of review<br>MA	To qualitatively and quantitatively summarize the evidence for the use of BONT-A injections in children and adolescents with migraine.	I: BONT-A<br><br>C: Any comparator or no control group	Disorder<br>CM (IHS)<br><br>N = 491<br>F = 70% to 100%<br><br>Age range<br>Children and adolescents ≤ 18 years)<br><br>Mean (range) age<br>15.5 years (14.7 to 16.7) years	6 (MEDLINE, EMBASE, PsycINFO, CINAHL, Plus, Cochrane Library<br><br>Date range<br>Inception to February 7, 2024	14 studies (12 obervational and 2 RCTs)<br><br>Country of origin<br>USA (10), Canada (1), Germany (1), Italy (1), Korea (1)	2009-2023	Primary studies<br>Data extraction tables<br><br>Quality assessment<br>American Academy of Neurology Risk of Bias grading scheme	Outcomes<br>1. Change of monthly headache episodes<br>2. Change of headache severity<br><br>Time frame<br>6 weeks to 28 months (mean 2-2.6 inj cycles, i.e. appr 6 months)	Data synthesis<br>Hedge's *g* (95% CI),<br><br>Random-effects model<br>Forest plots<br><br>Heterogeneity<br>τ^2 test and Higgins I^2 statistics<br><br>Publication bias<br>Funnel plots and Begg's test<br><br>$P < 0.05$
Shamliyan et al, 2013<br><br>Country<br>USA<br><br>Type of review<br>MA	To compare the effectiveness and safety of drugs for preventing migraine attacks in adults	I: BONT-A<br><br>C: Placebo	Disorder:<br>CM (IHS)<br><br>N = 285<br>F = 85%<br><br>Age range<br>Adults (18-65 years)	3 (MEDLINE, Cochrane, SCIRUS]<br><br>Date range<br>Until May 20, 2012	245 studies<br><br>16 studies on BONT-A<br><br>Country of origin<br>USA (7), North America (3), Chile	2000-2011	Primary studies<br>Data extraction table<br><br>Quality assessment<br>Cochrane Handbook for Systematic	Outcomes<br>1. ≥50% reduction of monthly migraine episodes<br>2. Adverse events<br>Time frame<br>6 months	Data synthesis<br>RR (95% CI)<br>NNTB (95% CI)<br>NNTH (95% CI)<br>Random effects model<br>Forest plots

			Mean (range) age<br>42.8 years (18 to 65 years)		(1), North America/ Europe (1), Europe (1), Germany (1)<br>India (1), Thailand (1)		Reviews of Interventions		Heterogeneity Chi^2 test and Higgins I^2 statistics<br><br>Publication bias<br>Assessed, but formal statistical test applied<br><br>Evidence for effect<br>Criteria based on published guidelines
Shen et al, 2020<br><br>Country<br>China<br><br>Type of review<br>MA	To investigate the impact of BONT-A as a therapeutic regimen for the management of adult migraine disorders	I: BONT-A<br><br>C: Placebo	Disorder<br>EM and CM<br><br>N = 6610<br>F = 70.7%<br><br>Age range<br>Adults (> 18 years)<br><br>Mean (range) age<br>43.5 years (18-65) years	3 (PubMed, EMBASE, Cochrane Library)<br><br>Date range<br>Inception to July 6, 2019	18 studies<br>RCTs<br><br>Country of origin<br>USA (9), Brazil (1), North America/ Europe (2), Europe (1), Germany (2), Italy (1), India (1), China (1)	2000-2015	Primary studies<br>Data extraction tables<br><br>Quality assessment<br>Cochrane Handbook	Outcomes<br>1. Changes of monthly headache episodes<br>2. ≥ 50% reduction of monthly headache episodes<br>3. HIT-6<br>4. MIDAS<br>5. Adverse events<br><br>Time frame<br>84-395 days	Data synthesis<br>RR (95% CI)<br>MD: (95% CI)<br><br>Random effects model<br>Forest plots<br><br>Heterogeneity<br>τ^2 test and Higgins I^2 statistics<br><br>Publication bias<br>Funnel plots
Shuhendler et al, 2009<br><br>Country<br>???<br><br>Type of review<br>MA	To assess the efficacy of BONT-A in lowering the frequency of migraine headaches in patients with episodic migraines	I: BONT-A<br><br>C: Placebo	Disorder<br>EM<br><br>N = 1601<br>F = 83.8%<br>Age range<br>Adults (> 18 years)<br><br>Mean (range) age<br>43 years (38.3-45.3) years	3 (PubMed, Google Scholar, Cochrane Library)<br>Date range<br>Inception to October 2007	8 studies<br>RCTs<br><br>Country of origin<br>USA (6), Europe (1), Germany (1)	2000-2007	Primary studies<br>Data extraction tables<br>Quality assessment<br>Downs and Black scale	Outcomes<br>1. Change from baseline to end point in monthly migraine episodes<br><br>Time frame<br>30 to 90 days	Data synthesis<br>SMD (95% CI)<br><br>Random effect model<br>Forest plots<br><br>Heterogeneity<br>Chi^2 test and Higgins I^2 statistics

									Publication bias<br>Not reported<br>$p < 0.05$
Zheng et ai, 2020<br><br>Country<br>China<br><br>Type of review<br>NMA	To perform a network meta-analysis to compare the effectiveness and acceptability between topiramate, acupuncture, and BONT-A	I: BONT-A, topiramate, acupuncture<br><br>C: Placebo	Disorder<br>CM<br><br>N = 2545<br>F = 76.3%<br><br>Age range<br>Adults (> 18 years)<br><br>Mean (range) age<br>38.2–48.8 years	5 (OVID MEDLINE, EMBASE, the Cochrane register of controlled trials (CENTRAL), the Chinese Clinical Trial Register, and clinicaltrials.gov<br><br>Date range<br>Inception to March 1, 2020	15 studies<br>RCTs<br><br>Country of origin<br>Global (1), USA (6), Brazil (1), Europe (1), the Netherlands (1), Italy (3), Iran (1), China (1)<br><br>10 studies<br>BONT-A	2003-2019	Primary studies<br>Data extraction tables<br><br>Quality assessment<br>Own method	Outcomes<br>1. Change of monthly headache days<br>2. Change of monthly migraine days<br>3. Change of headache intensity<br>4. Change of HIT-6<br>5. Change of MIDAS<br>6. Change of QoL<br>7. Adverse events<br><br>Time frame<br>12 and 24 weeks	Data synthesis<br>SMD (95% CI)<br>RR (95% CI)<br>Random effects Model<br>NMA and forest plots<br><br>Heterogeneity<br>Higgins I^2 statistics<br><br>Publication bias<br>Sensitivity analysis
Zhao et al, 2024<br><br>Country<br>China<br><br>Type of review<br>NMA	To investigate the effectiveness and safety of different drugs for the prophylaxis of CM based on multiple outcome measure	I: BONT-A or other prophylactics<br><br>C: Placebo	Disorder<br>CM (ICHD, ICHD-2, ICHD-3, ICHD-3β, IHS)<br><br>N = 8789<br>F = 75.7%<br>Age range<br>all<br><br>Mean (range) age<br>38.7 years (14.9 to 47.8) years	4 (PubMed, EMBASE, Cochrane Library, Web of Science)<br><br>Date range<br>Inception to August 1, 2023	24 studies<br><br>Country of origin<br>America (14), Brazil (1), UK (1), the Netherlands (1), Italy (2), Iran (1), India (1), China (2), Japan (1)<br><br>7 studies<br>BONT-A	2005-2022	Primary studies<br>Data extraction tables<br><br>Quality assessment<br>Cochrane bias risk assessment tool	Outcomes<br>1. Change of monthly migraine days<br>2.≥ 50% reduction of migraine days<br>3. Change of MIDAS<br>4. Adverse events<br><br>Time frame<br>Not reported	Data synthesis<br>WMD (95% CI)<br>RR (95% CI)<br><br>Bayesian framework-based random-effects model.<br>SUCRA for cumulative ranking probability<br><br>Publication bias<br>Funnel plots

BDI: Beck's depression index; BONT-A: botulinumtoxin; CM: Chronic migraine; EM: Episodic migraine; F: Females; GRADE: Grading of Recommendations Assessment, Development and Evaluation; ICHD: International classification of headache disorders; IHS: International headache society; HIT-&: Headache impact test; HIS: International headache society; MA: Meta-analysis; MD: Mean difference; MIDAS: Migraine disability assessment; MSQ: Migraine specific quality of life

questionnaire; NMA: Network meta-analysis; NNTB: The number needed to treat to benefit; NNTH: The number needed to treat to harm; p: p-value; PHQ-9: Patient history questionnaire-9; QoL: Quality of life; RR: Risk ratio; SUCRA: surface under the cumulative ranking curve; WMD: Weighted mean difference.

Table S4. Primary articles from the 14 meta-analyses included in the umbrella review

Primary article	Included in study (authors)
Ali SS, Bragin I, Rende E, Mejico L, Werner KE. Further evidence that onabotulinum toxin is a viable treatment option for pediatric chronic migraine patients. Cureus. 2016;11(3):e4343.	Lindsay et al 2024
Anand KS, Prasad A, Singh MM, Sharma S, Bala K. Botulinum toxin type A in prophylactic treatment of migraine. American Journal of Therapeutics. 2006;13:183-187.	Herd et al, 2018, Jackson et al, 2012, Shamliyan et al 2013, Shen et al 2020
Aurora SK, Gawel M, Brandes JL, Pokta S, Vandenburgh AM, Group BNAEMS. Botulinum toxin type a prophylactic treatment of episodic migraine: a randomized, double-blind, placebocontrolled exploratory study. Headache. 2007;47:486-499.	Bruloy et al 2018, Frank et al 2021, Herd et al, 2018, Jackson et al, 2012, Shamliyan et al 2013, Shen et al 2020, Shuhendler et al 2009
Aurora SK, Dodick DW, Turkel CC, DeGryse RE, Silberstein SD, Lipton RB, Diener HC, Brin MF. OnabotulinumtoxinA for treatment of chronic migraine: results from the doubleblind, randomized, placebo-controlled phase of the PREEMPT 1 trial. Cephalalgia. 2010;30:793–803.	Bruloy et al 2018, Chen et al 2021, Herd et al, 2018, Jackson et al 2012, Shamiliyan et al 2013, Shen et al 2020, Zheng et al 2020
Aurora SK, Winner P, Freeman MC, et al. OnabotulinumtoxinA for treatment of chronic migraine: pooled analyses of the 56-week PREEMPT clinical program. Headache. 2011;51:1358-1373.	Corasaniti et al 2023, Frank et al 2021
Aurora SK, Dodick DW, Diener H-C, et al. OnabotulinumtoxinA for chronic migraine: efficacy, safety, and tolerability in patients who received all five treatment cycles in the PREEMPT clinical program. Acta Neurol Scand. 2014;129:61-70.	Corasaniti et al 2023
Aydinlar EI, Dikmen PY, Kosak S, Kocaman AS. OnabotulinumtoxinA effectiveness on chronic migraine, negative emotional states and sleep quality: a single-center prospective cohort study. J Headache Pain. 2017;18:23.	Affatato et al 2021
Barrientos, N. and Chana, P. Botulinum toxin type A in prophylactic treatment of migraine headaches: a preliminary study. The Journal of Headache and Pain. 2003;4:146-151.	Bruloy et al 2018, Herd et al, 2018, Shamliyan et al 2013, Shen et al 2020, Shuhendler et al 2009
Bartolini M, Silvestrini M, Taffi R, Lanciotti C, Luconi R, Capecci M, Provinciali L. Efficacy of topiramate and valproate in chronic migraine. Clin Neuropharmacol. 2005;28:277–279.	Zhao et al 2024
Bernhard M, Bertsche A, Syrbe S, Weise S, Merkenschlager A. Botulinumtoxin-injektionen bei chronischer migräne im jugendalter– eine frühzeitige therapieoption in der transition von der neuropädiatrie zur neurologie. Fortschritte Neurol Psychiatr. 2014;82:39-42.	Lindsay et al 2024
Bigal ME, Dodick DW, Krymchantowski AV, VanderPluym JH, Tepper SJ, Aycardi E, Loupe PS, Ma Y, Goadsby PJ. TEV-48125 for the preventive treatment of chronic migraine: Efficacy at early time points. Neurology. 2016;87:41–48.	Zhao et al 2024
Blumenfeld AM, Schim JD, Chippendale TJ. Botulinum toxintype A and divalproex sodium for prophylactic treatment of episodic or chronic migraine. Headache. 2008;48:210-220.	Herd et al, 2018
Blumenfeld AM, Tepper SJ, Robbins LD, Manack Adams A, Buse DC, Orejudos A, Silberstein SD. Effects of onabotulinumtoxinA treatment for	Affatato et al 2021

chronic migraine on common comorbidities including depression and anxiety. J Neurol Neurosurg Psychiatry. 2019;90:353–360.	
Blumenkron D, Rivera C, Cuevas C. EKicacy of botulinum toxin type A in patients with migraine [Eficacia del tratamiento con toxina botulinica tipo A en pacientes con migrana]. Medicina Interna de Mexico. 2006;22:25-31.	Herd et al, 2018
Boudreau GP, Grosberg BM, McAllister PJ, Lipton RB, Buse DC. Prophylactic onabotulinumtoxinA in patients with chronic migraine and comorbid depression: An open-label, multicenter, pilot study of efficacy, safety and effect on headache-related disability, depression, and anxiety. Int J Gen Med. 2015;18:79–86.	Affatato et al 2021
Bragin I, Zidan A, Burke D, Mejico L, Ali S, Werner K. The use of botulinum toxin type A (BoNTA) injection in treatment of chronic migraine in pediatric practice. Cephalalgia. 2015;35(6_suppl):65.	Lindsay et al 2024
Cady R, Schreiber C. Botulinum toxin type A as migraine preventive treatment in patients previously failing oral prophylactic treatment due to compliance issues. Headache. 2008;48:900-913.	Herd et al, 2018, Jackson et al, 2012, Shamliyan et al 2013
Cady RK, Schreiber CP, Porter JA, Blumenfeld AM, Farmer KU. A multi-center double-blind pilot comparison ofonabotulinumtoxinA and topiramate for the prophylactic treatment of chronic migraine. Headache. 2011;51:21-32.	Bruloy et al 2018, Corasaniti et al 2023, Herd et al, 2018, Jackson et al 2012, Zheng et al 2020
Cady R, Turner I, Dexter K, Beach ME, Cady R, Durham P. An exploratory study of salivary calcitonin gene-related peptide levels relative to acute interventions and preventative treatment with onabotulinumtoxinA in chronic migraine. Headache. 2014;54:269-277.	Herd et al, 2018, Shen et al 2020
Chan VW, McCabe EJ, MacGregor DL. Botox treatment for migraine and chronic daily headache in adolescents. J Neurosci Nurs. 2009;41:235-243.	Lindsay et al 2024
Chankrachang S, Arayawichanont A, Poungvarin N, Nidhinandana S, Boonkongchuen P, Towanabut S, et al. Prophylactic botulinum type A toxin complex (Dysport) for migraine without aura. Headache. 2011;51:52-63.	Bruloy et al 2018, Herd et al, 2018, Jackson et al, 2012, Shamliyan et al 2013
Choi Y, Bae C. OnabotulinumtoxinA as one of the preventive treatments for chronic migraine in Korean adolescents. Cephalalgia. 2016;36:17-18.	Lindsay et al 2024
Chowdhury D, Bansal L, Duggal A, Datta D, Mundra A, Krishnan A, Koul A, Gupta A. TOP-PRO study: a randomized double- blind controlled trial of topiramate versus propranolol for prevention of chronic migraine. Cephalalgia. 2022;42:396–408.	Zhao et al 2024
Detke HC, Goadsby PJ, Wang S, Friedman DI, Selzler KJ, Aurora SK. Galcanezumab in chronic migraine: the randomized, double-blind, placebo-controlled REGAIN study. Neurology. 2018;91:e2211–e2221.	Chen et al 2021, Zhao et al 2024
Demiryurek BE, Ertem DH, Tekin A, Ceylan M, Aras YG, Gungen BD. Effects of onabotulinumtoxinA treatment on efficacy, depression, anxiety, and disability in Turkish patients with chronic migraine. Neurol Sci.2016;37:1779–1784.	Affatato et al 2021
Diener HC, Bussone G, Van Oene JC, Lahaye M, Schwalen S, Goadsby PJ, et al. Topiramate reduces headache days in chronic migraine: a randomized, double-blind, placebo-controlled study. Cephalalgia. 2007;27:814–823.	Zhao et al 2024, Zheng et al 2020
Diener HC, Dodick DW, Aurora SK, Turkel CC, DeGryse RE, Lipton RB, et al. OnabotulinumtoxinA for treatment of chronic migraine: results from the double-blind, randomized, placebo-controlled phase of the PREEMPT 2 trial. Cephalalgia. 2010;30:804-814.	Bruloy et al 2018, Chen et al 2021, Corasaniti et al 2023, Herd et al, 2018, Jackson et al 2012, Shamliyan et al 2013, Shen et al 2020, Zheng et al 2020,
Dodick DW, Silberstein S, Saper J, et al. The impact of topiramate on health-related quality of life indicators in chronic migraine. Headache. 2007;47:1398-1408.	Zheng et al 2020

Dodick DW, Lipton RB, Silberstein S, Goadsby PJ, Biondi D, Hirman J, Cady R, Smith J. Eptinezumab for prevention of chronic migraine: a randomized phase 2b clinical trial. Cephalalgia. 2019;39:1075–1085.	Chen et al 2021, Zhao et al 2024
Elkind AH, O'Carroll P, Blumenfeld A, DeGryse R, Dimitrova R, BoNTASG. A series of three sequential, randomized, controlled studies of repeated treatments with botulinum toxin type A for migraine prophylaxis. Journal of Pain. 2006;7:688-696.	Bruloy et al 2018, Herd et al, 2018, Jackson et al, 2012, Shamliyan et al 2013, Shen et al 2020, Shuhendler et al 2009
Evers S, Vollmer-Haase J, Schwaag S, Rahmann A, Husstedt IW, Frese A. (2004) Botulinum toxin A in the prophylactic treatment of migraine--a randomized, double-blind, placebo-controlled study. Cephalalgia. 2004;24:838-843.	Bruloy et al 2018, Frank et al 2021, Jackson et al 2012, Shen et al 2020
Ford J, Tassorelli C, Leroux E, Wang S, Ayer D, Nichols R, Detke H. Changes in patient functioning and disability: results from a phase 3, double-blind, randomized, placebo-controlled clinical trial evaluating galcanezumab for chronic migraine prevention (REGAIN). Qual Life Res. 2021;30:105–115.	Zhao et al 2024
Freitag FG, Diamond S, Diamond M, Urban G. Botulinum toxin type A in the treatment of chronic migraine without medication overuse. Headache. 2008;48:201-209.	Bruloy et al 2018, Chen et al 2021, Frank et al 2021, Herd et al, 2018, Jackson et al 2012, Shamliyan et al 2013, Shen et al 2020, Zhao et al 2024, Zheng et al 2020
Guerzoni S, Pellesi L, Baraldi C, Pini LA. Increased efficacy of regularly repeated cycles with OnabotulinumtoxinA in MOH patients beyond the first year of treatment. J Headache Pain. 2015;17:48.	Affatato et al 2021
Hollanda L, Monteiro L, Melo AS. Botulinum toxin type A for cephalic cutaneous allodynia in chronic migraine: a randomized, double-blinded, placebo-controlled trial. Neurology International. 2014;6(4):70-73.	Bruloy et al 2018, Chen et al 2021, Herd et al, 2018, Zhao et al 2024, Zheng et al 2020
Hou M, Xie JF, Kong XP, Zhang Y, Shao YF, Wang C, et al. Acupoint injection of onabotulinumtoxin A for migraines. Toxins. 2015;7:4442-454.	Bruloy et al 2018, Herd et al, 2018, Shen et al 2020
Jabbari B. Investigation of efficacy and safety of botulinum toxin A (BOTOX-Allergan Inc.) in migraine headaches. clinicaltrials.gov/ct2/show/NCT00660192 (first received 17 April 2008).	Herd et al, 2018
Jost WH. Low-dosed botulinum toxin A in the prophylactic management of unilateral migraine: a randomized doubleblind placebo-controlled crossover study. Open Pain Journal. 2011;4:4-7.	Herd et al, 2018
Kabbouche M, O'Brien H, Hershey AD. OnabotulinumtoxinA in pediatric chronic daily headache. Curr Neurol Neurosci Rep. 2012;12:114-117.	Lindsay et al 2024
Karian V, Morton H, Schefter ZJ, et al. OnabotulinumtoxinA for pediatric migraine. Pain Manag Nurs. 2023;24(6):610-616.	Lindsay et al 2024
Kennedy G. Longer term outcomes for patients with chronic migraine treated with OnabotulinumtoxinA BOTOX and implications for a Headache Service: Real-life data for 120 patients treated at Sunderland Royal Hospital, UK. Cephalalgia 2017;37:333.	Lanteri-Minet et al 2022
Kollewe K, Escher CM, Wulff DU, Fathi D, Paracka L, Mohammadi B, Karst M, Dressler D. Long-term treatment of chronic migraine with OnabotulinumtoxinA: efficacy, quality of life and tolerability in a real-life setting. J Neural Transm. 2016;123:533–540.	Affatato et al 2021
Lai KL, Niddam DM, Fuh JL, Chen SP, Wang YF, Chen WT, Wu JC, Wang SJ. Flunarizine versus topiramate for chronic migraine prophylaxis: a randomized trial. Acta Neurol Scand .2017;135:476–483.	Zhao et al 2024
Lauretti GR, Rosa CP, Kitayama A, Lopes BCP. Comparison of Botox or Prosigne and facial nerve blockade as adjuvant in chronic migraine. Journal of Biomedical Science and Engineering. 2014;7:446-452.	Herd et al, 2018, Shen et al 2020

Lee MJ, Lee C, Choi H, et al. Factors associated with favorable outcome in botulinum toxin A treatment for chronic migraine: A clinic-based prospective study. J Neurol Sci. 2016;363:51–54.	Lanteri-Minet et al 2022
Lin K-H, Chen S-P, Fuh J-L, et al. Efficacy, safety, and predictors of response to botulinum toxin type A in refractory chronic migraine: A retrospective study. J Chin Med Assoc. 2014;77:10–15.	Lanteri-Minet et al 2022
Lipton RB, Varon SF, Grosberg B, et al. OnabotulinumtoxinA improves quality of life and reduces impact of chronic migraine. Neurology. 2011;77:1465-1472.	Shamliyan et al 2013
Lipton RB, Rosen NL, Ailani J, DeGryse RE, Gillard PJ, Varon SF. OnabotulinumtoxinA improves quality of life and reduces impact of chronic migraine over one year of treatment: Pooled results from the PREEMPT randomized clinical trial program. Cephalalgia. 2016;36:899-908.	Shen et al 2020
Lipton RB, Goadsby PJ, Smith J, Schaeffler BA, Biondi DM, Hirman J, Pederson S, Allan B, Cady R. Efficacy and safety of eptinezumab in patients with chronic migraine: PROMISE-2. Neurology. 2020;94:e1365–e1377.	Zhao et al 2024
Maasumi K, Thompson NR, Kriegler JS, Tepper SJ. Effect of OnabotulinumtoxinA Injection on Depression in Chronic Migraine. Headache. 2015;55:1218–1222.	Affatato et al 2021
Magalhaes E, Menezes C, Cardeal M, Melo A. Botulinum toxin type A versus amitriptyline for the treatment of chronic daily migraine. Clin Neurol Neurosurg. 2010;112:463-466.	Jackson et al 2012
Matharu M, Halker R, Pozo-Rosich P, DeGryse R, Manack, Adams A, Aurora SK. (2017) The impact of onabotulinumtoxinA on severe headache days: PREEMPT 56-week pooled analysis. The Journal of Headache and Pain 2017; 8,78	Shen et al 2020
Mathew NT, Frishberg BM, Gawel M, Dimitrova R, Gibson J, Turkel C, et al. Botulinum toxin type A (BOTOX) for the prophylactic treatment of chronic daily headache: a randomized, double-blind, placebo-controlled trial. Headache. 2005;45:293-307.	Jackson et al 2012, Shamliyan et al 2013
Mathew NT, JaKri SF. A double-blind comparison of onabotulinumtoxinA (BOTOX) and topiramate (TOPAMAX) for the prophylactic treatment of chronic migraine: a pilot study. Headache. 2009;49:1466-7148.	Corasaniti et al 2023, Zheng et al 2020
Mei D, Ferraro D, Zelano G, Capuano A, Vollono C, Gabriele C, Di Trapani G. Topiramate and triptans revert chronic migraine with medication overuse to episodic migraine. Clin Neuropharmacol. 2006;29:269–275.	Zhao et al 2024, Zheng et al 2020
Naderinabi B, Saberi A, Hashemi M, Haghighi M, Biazar G, Abolhasan Gharehdaghi F, Sedighinejad A, Chavoshi T. Acupuncture and botulinum toxin A injection in the treatment of chronic migraine: a randomized controlled study. Caspian J Intern Med. 2017;8:196–204.	Corasaniti et al 2023, Zhao et al 2024, Zheng et al 2020
Navarrete Perez JJ. Wearing-off effect of onabotulinumtoxinA in chronic migraine: Evaluation in a series of 117 patients. Eur J Neurol. 2017;24:547.	Lanteri-Minet et al 2022
Negro A, Curto M, Lionetto L, et al. A two years openlabel prospective study of OnabotulinumtoxinA 195 U in medication overuse headache: A real-world experience. J Headache Pain. 2016;17:1–9.	Lanteri-Minet et al 2022
O'Brien H, Kabbouche M, Kacperski J, et al. Treatment of chronic migraine using onabotulinumtoxinA may be less effective in pediatric patients with history of depression or anxiety. Headache. 2014;54(S1):45.	Lindsay et al 2024
Ondo WG, Vuong KD, Derman HS. Botulinum toxin A for chronic daily headache: a randomized, placebo-controlled, parallel design study. Cephalalgia. 2004;24:60-65.	Chen et al 2021, Jackson et al 2012, Zheng et al 2020

Papetti L, Frattale I, Ursitti F, et al. Real life data on onabotulinumtoxinA for treatment of chronic migraine in pediatric age. J Clin Med. 2023;12:1802.	Lindsay et al 2024
Pijpers JA, Kies DA, Louter MA, van Zwet EW, Ferrari MD, Terwindt GM. Acute withdrawal and botulinum toxin A in chronic migraine with medication overuse: a double-blind randomized controlled trial. Brain. 2019;42:1203–1214.	Zhao et al 2024, Zheng et al 2020
Pedraza MI. OnabotulinumtoxinA treatment for chronic migraine: experience in 52 patients treated with the PREEMPT paradigm. SpringerPlus. 2015;4.	Lanteri-Minet et al 2022
Petri S, Tölle T, Straube A, PfaKenrath V, Stefenelli U, Ceballos-Baumann A, et al. Botulinum toxin as preventive treatment for migraine: a randomized double-blind study. European Neurology. 2009;62:204-211.	Bruloy et al 2018, Frank et al 2021, Jackson, Shamliyan et al 2013
Quintas S, Garcia-Azorin D, Heredia P, et al. Wearing off response to onabotulinumtoxinA in chronic migraine: Analysis in a series of 193 patients. Pain Medicine. 2019; 20: 1815–1821.	Lanteri-Minet et al 2022
Ranoux D, Martin e G, Espagne-Dubreuilh G, Amilhaud-Bordier M, Caire F, Magy L. OnabotulinumtoxinA injections in chronic migraine, targeted to sites of pericranial myofascial pain: an observational, open label, real-life cohort study. J Headache Pain. 2017;18:75.	Lanteri-Minet et al 2022
Relja M, Poole AC, Schoenen J, Pascual J, Lei X, Thompson C, et al. A multicentre, double-blind, randomized, placebocontrolled, parallel group study of multiple treatments of botulinum toxin type A (BoNTA) for the prophylaxis of episodic migraine headaches. Cephalalgia. 2007;27:492-503.	Bruloy et al 2018, Frank et al 2021, Frank et al 2021, Jackson et al 2012, Shamliyan et al 2013, Shen et al 2020, Shuhendler et al 2009
Romoli M, Corbelli I, Bernetti L, et al. Stopping Onabotulinum treatment after the first 2 cycles might not be justified: Results of a real-life monocentric prospective study in chronic migraine. J Headache Pain. 2017;18.	Lanteri-Minet et al 2022
Rothrock JF, Adams AM, Lipton RB, Silberstein SD, Jo E, Zhao X, Blumenfeld AM. FORWARD study: evaluating the comparative effectiveness of OnabotulinumtoxinA and topiramate for headache prevention in adults with chronic migraine. Headache. 2019;59:1700–1713.	Corasaniti et al 2023, Zhao et al 2024
Russo M, Manzoni GC, Taga A, Genovese A, Veronesi L, Pasquarella C, Sansebastiano GE, Torelli P. The use of onabotulinum toxin A (Botox(®)) in the treatment of chronic migraine at the Parma Headache Centre: a prospective observational study. Neurol Sci. 2016;37:1127–1131.	Affatato et al 2021, Lanteri-Minet et al 2022
Sakai F, Suzuki N, Kim BK, Igarashi H, Hirata K, Takeshima T, Ning X, Shima T, Ishida M, Iba K, Kondo H, Koga N. Efficacy and safety of fremanezumab for chronic migraine prevention: multicenter, randomized, double-blind, placebo-controlled, parallel-group trial in Japanese and Korean patients. Headache. 2021;61:1092–1101.	Zhao et al 2024
Sandrini G, Perrotta A, Tassorelli C, et al. Botulinum toxin type-A in the prophylactic treatment of medication-overuse headache: a multicenter, double-blind, randomized, placebo-controlled, parallel group study. J Headache Pain. 2011;12:427-433.	Bruloy et al 2018, Chen et al 2021, Jackson et al 2012, Shen et al 2020, Zheng et al 2020
Santana L, Liu C. Experience of onabotulinumtoxinA A injections for chronic migraine headaches in a pediatric chronic pain clinic. J Pediatr Pharmacol Ther. 2021;26:151-156.	Lindsay et al 2024
Santoro A, Copetti M, Miscio AM, et al. Chronic migraine long-term regular treatment with onabotulinumtoxinA: a retrospective real-life observational study up to 4 years of therapy. Neurol Sci. 2020.	Lanteri-Minet et al 2022
Sanz AC. Experience with botulinum toxin in chronic migraine. Neurologia. 2018;33:499–504.	Lanteri-Minet et al 2022
Saper JR, Mathew NT, Loder EW, DeGryse R, VanDenburgh AM, BoNTASG. A double-blind, randomized, placebo-controlled comparison of	Bruloy et al 2018, Jackson et al 2012, Shamliyan et al 2013, Shen et al 2020, Shuhendler et al 2009

botulinum toxin type a injection sites and doses in the prevention of episodic migraine. Pain Medicine. 2007;8:478-85.	
Sarchielli P, Romoli M, Corbelli, et al. Stopping onabotulinum treatment after the first two cycles might not be justified: Results of a real-life monocentric prospective study in chronic migraine. Front Neurol. 2017;8:655.	Lanteri-Minet et al 2022
Shah S, Calderon MD, Wu WD, Grant J, Rinehart J. Onabotulinumtoxin A (BOTOX®) for prophylactic treatment of pediatric migraine: a retrospective longitudinal analysis. J Child Neurol. 2018;33:580-586.	Lindsay et al 2024
Shah S, Calderon MD, Crain N, Pham J, Rinehart J. Effectiveness of onabotulinumtoxinA (BOTOX) in pediatric patients experiencing migraines: a randomized, double-blinded, placebo-controlled crossover study in the pediatric pain population. Reg Anesth Pain Med. 2021;46:41-48.	Lindsay et al 2024
Shehata HS, Esmail EH, Abdelalim A, El-Jaafary S, Elmazny A, Sabbah A, Shalaby NM. Repetitive transcranial magnetic stimulation versus botulinum toxin injection in chronic migraine prophylaxis: A pilot randomized trial. J. Pain Res. 2016;9:771–777.	Corasaniti et al 2023
Silberstein S, Mathew N, Saper J, Jenkins S. Botulinum toxin type A as a migraine preventive treatment. Headache. 2000;40(6):445-450.	Bruloy et al 2018, Frank et al 2021, Jackson et al 2012, Shamliyan et al 2013, Shen et al 2020, Shuhendler et al 2009
Silberstein SD, Stark SR, Lucas SM, Christie SN, Degryse RE, Turkel CC, et al. Botulinum toxin type A for the prophylactic treatment of chronic daily headache: a randomized, doubleblind, placebo-controlled trial. Mayo Clinic Proceedings. 2005;80:1126-1137.	Jackson et al 2012, Shamliyan et al 2013
Silberstein SD, Lipton RB, Dodick DW, Freitag FG, Ramadan N, Mathew N, et al. EKicacy and safety of topiramate for the treatment of chronic migraine: a randomized, double-blind, placebo-controlled trial. Headache. 2007;47:170-180.	Zhao et al 2024, Zheng et al 2020
Silberstein S, Lipton R, Dodick D, Freitag F, Mathew N, Brandes J, Bigal M, Ascher S, Morein J, Wright P, Greenberg S, Hulihan J. Topiramate treatment of chronic migraine: a randomized, placebo-controlled trial of quality of life and other efficacy measures. Headache. 2009;49:1153–1162.	Zhao et al 2024, Zheng et al 2020
Silberstein SD, Dodick DW, Bigal ME, Yeung PP, Goadsby PJ, Blankenbiller T, et al. Fremanezumab for the preventive treatment of chronic migraine. New England Journal of Medicine. 2017;377:2113-2122.	Chen et al 2021, Zhao et al 2024
Silberstein S, Diamond M, Hindiyeh NA, Biondi DM, Cady R, Hirman J, Allan B, Pederson S, Schaeffler B, Smith J. Eptinezumab for the prevention of chronic migraine: efficacy and safety through 24 weeks of treatment in the phase 3 PROMISE-2 (Prevention of migraine via intravenous ALD403 safety and efficacy-2) study. J Headache Pain. 2020;21:120.	Zhao et al 2024
Silvestrini M, Bartolini M, Coccia M, Baruffaldi R, Taffi R, Provinciali L. Topiramate in the treatment of chronic migraine. Cephalalgia. 2003;23:820-824.	Zheng et al 2020
Stark C. Real-world effectiveness of onabotulinumtoxinA treatment for the prevention of headaches in adults with chronic migraine in Australia: A retrospective study. J Headache Pain 2019;20.	Lanteri-Minet et al 2022
Taddei-Allen P, Deleon L, Oelofsen M, Page N. Comparing patient-reported outcomes in patients using CGRP antagonists or onabotulinumtoxinA for chronic migraine. J Managed Care Spec Pharm. 2019;25:S60.	Lanteri-Minet et al 2022
Tepper S, Ashina M, Reuter U, Brandes JL, Doležil D, Silberstein S, Winner P, Leonardi D, Mikol D, Lenz R. Safety and efficacy of erenumab for preventive	Zhao et al 2024

treatment of chronic migraine: a randomised, double-blind, placebo-controlled phase 2 trial. Lancet Neurol. 2017;16:425–434	
Torres-Ferrus M, Gallardo VJ, Alpuente A, et al. Influence of headache pain intensity and frequency on migraine-related disability in chronic migraine patients treated with OnabotulinumtoxinA. J Headache Pain. 2020;21:1–8.	Lanteri-Minet et al 2022
Velasco-Juanes F. Clinical treatment of chronic and episodic migraine with onabotulinumtoxinA in a real-world setting. Drugs Therapy Persp. 2018;34:335–343.	Lanteri-Minet et al 2022
Vernieri F, Paolucci M, Altamura C, et al. Onabotulinumtoxin-A in chronic migraine: should timing and definition of non-responder status be revised? Suggestions from a real-life Italian multicenter experience. Headache. 2019;59:1300–1309.	Lanteri-Minet et al 2022
Vo AH, Satori R, Jabbari B, Green J, Killgore WD, Labutta R, et al. Botulinum toxin type-a in the prevention of migraine: a double-blind controlled trial. Aviation Space and Environmental Medicine. 2007;78(Suppl 5):B113-118.	Bruloy et al 2018, Jackson et al 2012, Shamliyan et al 2013, Shen et al 2020, Shuhendler et al 2009
Winner PK, Kabbouche M, Yonker M, Wangsadipura V, Lum A, Brin MF. A randomized trial to evaluate onabotulinumtoxinA for prevention of headaches in adolescents with chronic migraine. Headache. 2020;60:564-575.	Corasaniti et al 2023, Zhao et al 2024, Lindsay et al 2024
Yang C-P, Chang M-H, Liu P-E, et al. Acupuncture versus topiramate in chronic migraine prophylaxis: a randomized clinical trial. Cephalalgia. 2011;31:1510-1521.	Zheng et al 2020,
Yonker M, Marian M. Effectiveness of prophylactic onabotulinumtoxin A in adolescents with chronic migraine. Headache. 2016;56(S1):31-32.	Lindsay et al 2024
Yu S, Kim BK, Wang H, Zhou J, Wan Q, Yu T, Lian Y, Arkuszewski M, Ecochard L, Wen S, Yin F, Li Z, Su W, Wang SJ. A phase 3, randomised, placebo-controlled study of erenumab for the prevention of chronic migraine in patients from Asia: the DRAGON study. J Headache Pain. 2022;23:146.	Zhao et al 2024

Table S5. Preferred Reporting Items for Systematic reviews and Meta-Analyses (PRISMA) Checklist.

Section and Topic	Item #	Checklist item	Location where item is reported
**TITLE**			
Title	1	Identify the report as am umbrella review.	1
**ABSTRACT**			
Abstract	2	See the PRISMA 2020 for Abstracts checklist.	1
**INTRODUCTION**			
Rationale	3	Describe the rationale for the review in the context of existing knowledge.	2-3
Objectives	4	Provide an explicit statement of the objective(s) or question(s) the review addresses.	3
**METHODS**			
Eligibility criteria	5	Specify the inclusion and exclusion criteria for the review and how studies were grouped for the syntheses.	16-17
Information sources	6	Specify all databases, registers, websites, organisations, reference lists and other sources searched or consulted to identify studies. Specify the date when each source was last searched or consulted.	17
Search strategy	7	Present the full search strategies for all databases, registers and websites, including any filters and limits used.	Table S5
Selection process	8	Specify the methods used to decide whether a study met the inclusion criteria of the review, including how many reviewers screened each record and each report retrieved, whether they worked independently, and if applicable, details of automation tools used in the process.	17
Data collection process	9	Specify the methods used to collect data from reports, including how many reviewers collected data from each report, whether they worked independently, any processes for obtaining or confirming data from study investigators, and if applicable, details of automation tools used in the process.	17-18
Data items	10a	List and define all outcomes for which data were sought. Specify whether all results that were compatible with each outcome domain in each study were sought (e.g. for all measures, time points, analyses), and if not, the methods used to decide which results to collect.	16
	10b	List and define all other variables for which data were sought (e.g. participant and intervention characteristics, funding sources). Describe any assumptions made about any missing or unclear information.	18
Study risk of bias assessment	11	Specify the methods used to assess risk of bias in the included studies, including details of the tool(s) used, how many reviewers assessed each study and whether they worked independently, and if applicable, details of automation tools used in the process.	17
Effect measures	12	Specify for each outcome the effect measure(s) (e.g. risk ratio, mean difference) used in the synthesis or presentation of results.	Table S3
Synthesis methods	13a	Describe the processes used to decide which studies were eligible for each synthesis (e.g. tabulating the study intervention characteristics and comparing against the planned groups for each synthesis (item #5)).	18
	13b	Describe any methods required to prepare the data for presentation or synthesis, such as handling of missing summary statistics, or data conversions.	N/A
	13c	Describe any methods used to tabulate or visually display results of individual studies and syntheses.	18
	13d	Describe any methods used to synthesize results and provide a rationale for the choice(s). If meta-analysis was performed, describe the model(s), method(s) to identify the presence and extent of statistical heterogeneity, and software package(s) used.	18

Section and Topic	Item #	Checklist item	Location where item is reported
	13e	Describe any methods used to explore possible causes of heterogeneity among study results (e.g. subgroup analysis, meta-regression).	N/A
	13f	Describe any sensitivity analyses conducted to assess robustness of the synthesized results.	N/A
Reporting bias assessment	14	Describe any methods used to assess risk of bias due to missing results in a synthesis (arising from reporting biases).	N/A
Certainty assessment	15	Describe any methods used to assess certainty (or confidence) in the body of evidence for an outcome.	N/A
**RESULTS**			
Study selection	16a	Describe the results of the search and selection process, from the number of records identified in the search to the number of studies included in the review, ideally using a flow diagram.	3
	16b	Cite studies that might appear to meet the inclusion criteria, but which were excluded, and explain why they were excluded.	3-4, Tables S1, S2
Study characteristics	17	Cite each included study and present its characteristics.	3-14, Table S3
Risk of bias in studies	18	Present assessments of risk of bias for each included study.	Table S1
Results of individual studies	19	For all outcomes, present, for each study: (a) summary statistics for each group (where appropriate) and (b) an effect estimate, and its precision (e.g. confidence/credible interval), ideally using structured tables or plots.	Table S3
Results of syntheses	20a	For each synthesis, briefly summarise the characteristics and risk of bias among contributing studies.	13-14
	20b	Present results of all statistical syntheses conducted. If meta-analysis was done, present for each the summary estimate and its precision (e.g. confidence/credible interval) and measures of statistical heterogeneity. If comparing groups, describe the direction of the effect.	N/A
	20c	Present results of all investigations of possible causes of heterogeneity among study results.	N/A
	20d	Present results of all sensitivity analyses conducted to assess the robustness of the synthesized results.	N/A
Reporting biases	21	Present assessments of risk of bias due to missing results (arising from reporting biases) for each synthesis assessed.	N/A
Certainty of evidence	22	Present assessments of certainty (or confidence) in the body of evidence for each outcome assessed.	N/A
**DISCUSSION**			
Discussion	23a	Provide a general interpretation of the results in the context of other evidence.	14-15
	23b	Discuss any limitations of the evidence included in the review.	16
	23c	Discuss any limitations of the review processes used.	16
	23d	Discuss implications of the results for practice, policy, and future research.	16
**OTHER INFORMATION**			
Registration and protocol	24a	Provide registration information for the review, including register name and registration number, or state that the review was not registered.	16
	24b	Indicate where the review protocol can be accessed, or state that a protocol was not prepared.	16
	24c	Describe and explain any amendments to information provided at registration or in the	16

Section and Topic	Item #	Checklist item	Location where item is reported
		protocol.	
Support	25	Describe sources of financial or non-financial support for the review, and the role of the funders or sponsors in the review.	18
Competing interests	26	Declare any competing interests of review authors.	18
Availability of data, code and other materials	27	Report which of the following are publicly available and where they can be found: template data collection forms; data extracted from included studies; data used for all analyses; analytic code; any other materials used in the review.	18

From: Page MJ, McKenzie JE, Bossuyt PM, Boutron I, Hoffmann TC, Mulrow CD, et al. The PRISMA 2020 statement: an updated guideline for reporting systematic reviews. BMJ 2021;372:n71. doi: 10.1136/bmj.n71. This work is licensed under CC BY 4.0. To view a copy of this license, visit https://creativecommons.org/licenses/by/4.0/

Table S6. Search strategies for each database: 1) Medline; 2) Embase; 3) Cochrane; 4) Web of Science Core Collection; 5) Cinahl.

1. Medline

Interface: **Ovid MEDLINE(R) ALL** content coverage from 1946<br><br>Date of Search: 20 August 2024<br><br>Number of hits: 2,307<br><br>Comment: In Ovid, two or more words are automatically searched as phrases; i.e. no quotation marks are needed	Field labels<br><br>• exp/ = exploded MeSH term<br>• / = non exploded MeSH term<br>• .ti,ab,kf. = title, abstract and author keywords<br>• adjx = within x words, regardless of order<br>• * = truncation of word for alternate endings<br>• ? = 0-1 letter/number<br>• # = 1 letter/number

Database(s): **Ovid MEDLINE(R) ALL** 1946 to August 15, 2024
Search Strategy:

#	Searches	Results
1	Headache/	32614
2	exp Headache disorders/	41759
3	(migraine* or headache* or cephalalgia* or cephalgia* or sunct* or suna or hemicrania*).ti,ab,kf.	132815
4	or/1-3	144331
5	exp Botulinum Toxins/	19284
6	(abobotulinum or abobotulinumtoxin* or alluzience or azzalure or bocouture or boe-tox or bont a or bont serotype a or botox or botulin or botulinium or botulinum or BoNT-Aa or BoNT-A or cunox or daxibotulinum or daxibotulinumtoxin* or daxxify or dysport or evabotulinum or evabotulinumtoxin* or evosyal or gemibotulinum* or hutox or incobotulinum or incobotulinumtoxin* or jeuveau or letibotulinum or letibotulinumtoxin* or letybo or liztox or meditoxin or nabota or neuronox or nivobotulinum or nivobotulinumtoxin* or nuceiva or oculinum or onabotulinum or onabotulinumtoxin* or onaclostox or prabotulinum or prabotulinumtoxin* or prosigne or purtox or relabotulinum or relabotulinumtoxin* or reloxin or vistabel or vistabex or xeomeen or xeomin).ti,ab,kf.	27758
7	or/5-6	29966
8	4 and 7	1543
9	exp Migraine disorders/	33164
10	migraine*.ti,ab,kf.	44864
11	9 or 10	49304
12	("prevention and control" or drug therapy).fs.	2724356
13	(prevent* or treatment* or therapy or therapies).ti,ab,kf.	8433981
14	12 or 13	9428254
15	((systemati* or umbrella) adj3 review).ti.	270769
16	(meta-analy* or metaanaly* or metasynthe* or meta-synthe*).ti.	214553
17	(meta analysis or systematic review).pt.	358215
18	or/15-17	447984

19	11 and 14 and 18	1028
20	8 or 19	2513
21	limit 20 to (danish or english or greek or norwegian or portuguese or spanish or swedish)	2417
22	limit 21 to (editorial or letter)	110
23	21 not 22	2307

2. Embase

Interface: **embase.com** content coverage from 1947<br><br>Date of Search: 20 August 2024<br><br>Number of hits: 2,652<br><br>Comment: Emtree is the controlled vocabulary in Embase	Field labels<br><br>• /exp = exploded Emtree term<br>• /de = non exploded Emtree term<br>• ti,ab,kw = title, abstract and author keywords<br>• NEAR/x = within x words, regardless of order<br>• * = truncation of word for alternate endings<br>• # = 0-1 letter/number<br>• ? = 1 letter/number

#25	#23 NOT #24	2652
#24	(#12 OR #22) AND ([conference abstract]/lim OR [conference review]/lim OR [editorial]/lim OR [letter]/lim)	582
#23	#12 OR #22	3234
#22	(#16 OR #20) AND ([danish]/lim OR [english]/lim OR [greek]/lim OR [portuguese]/lim OR [spanish]/lim OR [swedish]/lim OR [norwegian]/lim)	1866
#21	#16 OR #20	1922
#20	#15 AND #19	879
#19	#17 OR #18	433859
#18	'meta analy*':ti OR metaanaly*:ti OR metasynthe*:ti OR 'meta synthe*':ti	258135
#17	((systemati* OR umbrella) NEAR/3 review):ti	312537

#16	#13 AND #14 AND ([systematic review]/lim OR [meta analysis]/lim)	1836
#15	#13 AND #14	36095
#14	prevent*:ti,ab,kw OR treatment*:ti,ab,kw OR therapy:ti,ab,kw OR therapies:ti,ab,kw	11606322
#13	'migraine'/exp/mj OR 'migraine*':ti,ab,kw	75977
#12	#9 NOT #10 AND ([danish]/lim OR [english]/lim OR [greek]/lim OR [portuguese]/lim OR [spanish]/lim OR [swedish]/lim OR [norwegian]/lim)	1437
#11	#9 NOT #10	1590
#10	#4 AND #8 AND ([conference abstract]/lim OR [conference review]/lim OR [editorial]/lim OR [letter]/lim)	1350
#9	#4 AND #8	2940
#8	#5 OR #6 OR #7	42186
#7	abobotulinum:ti,ab,kw OR abobotulinumtoxin*:ti,ab,kw OR alluzience:ti,ab,kw OR azzalure:ti,ab,kw OR bocouture:ti,ab,kw OR 'boe tox':ti,ab,kw OR 'bont a':ti,ab,kw OR 'bont serotype a':ti,ab,kw OR botox:ti,ab,kw OR botulin:ti,ab,kw OR botulinium:ti,ab,kw OR botulinum:ti,ab,kw OR BoNT-Aa:ti,ab,kw OR BoNT-A:ti,ab,kw OR cunox:ti,ab,kw OR daxibotulinum:ti,ab,kw OR daxibotulinumtoxin*:ti,ab,kw OR daxxify:ti,ab,kw OR dysport:ti,ab,kw OR evabotulinum:ti,ab,kw OR evabotulinumtoxin*:ti,ab,kw OR evosyal:ti,ab,kw OR gemibotulinum*:ti,ab,kw OR hutox:ti,ab,kw OR incobotulinum:ti,ab,kw OR incobotulinumtoxin*:ti,ab,kw OR jeuveau:ti,ab,kw OR letibotulinum:ti,ab,kw OR letibotulinumtoxin*:ti,ab,kw OR letybo:ti,ab,kw OR liztox:ti,ab,kw OR meditoxin:ti,ab,kw OR nabota:ti,ab,kw OR neuronox:ti,ab,kw OR nivobotulinum:ti,ab,kw OR nivobotulinumtoxin*:ti,ab,kw OR nuceiva:ti,ab,kw OR oculinum:ti,ab,kw OR onabotulinum:ti,ab,kw OR onabotulinumtoxin*:ti,ab,kw OR onaclostox:ti,ab,kw OR prabotulinum:ti,ab,kw OR prabotulinumtoxin*:ti,ab,kw OR prosigne:ti,ab,kw OR purtox:ti,ab,kw OR relabotulinum:ti,ab,kw OR relabotulinumtoxin*:ti,ab,kw OR reloxin:ti,ab,kw OR vistabel:ti,ab,kw OR vistabex:ti,ab,kw OR xeomeen:ti,ab,kw OR xeomin:ti,ab,kw	40922
#6	'botulinum toxin a'/mj	15284
#5	'botulinum toxin'/mj	8598

#4	#1 OR #2 OR #3	225598
#3	migraine*:ti,ab,kw OR headache*:ti,ab,kw OR cephalalgia*:ti,ab,kw OR cephalgia*:ti,ab,kw OR sunct*:ti,ab,kw OR suna:ti,ab,kw OR hemicrania*:ti,ab,kw	214258
#2	'cervicogenic headache'/mj OR 'chronic daily headache'/exp/mj OR 'cough headache'/exp/mj OR 'drug induced headache'/exp/mj OR 'exertional headache'/mj OR 'migraine'/exp/mj OR 'postdural puncture headache'/exp/mj OR 'posttraumatic headache'/mj OR 'postural headache'/mj OR 'sinus headache'/mj OR 'stabbing headache'/mj OR 'tension headache'/exp/mj OR 'throbbing headache'/mj OR 'thunderclap headache'/mj OR 'trigeminal autonomic cephalalgia'/exp/mj OR 'vascular headache'/mj	57794
#1	'headache'/exp/mj	38322

**3. Cochrane Library**	
Interface: **Wiley** content coverage: - Cochrane Database of Systematic Reviews - April 1996<br>Central Trials - Current content July 1998<br><br>Date of Search: 20 August 2024<br><br>Number of hits: 662	Field labels<br><br>• mh = exploded MeSH term<br>• mh ^= non exploded MeSH term<br>• ti,ab,kw = title, abstract and author keywords<br>• NEAR/x = within x words, regardless of order<br>• NEXT = used for truncated phrases<br>• NEXT/x = fixed word order<br>• * = truncation of word for alternate endings<br>• ? = 0-1 letter/number

ID Search Hits

#1 [mh ^Headache] 3205

#2 [mh "Headache disorders"] 4927

#3 (migraine*:ti,ab,kw OR headache*:ti,ab,kw OR cephalalgia*:ti,ab,kw OR cephalgia*:ti,ab,kw OR sunct*:ti,ab,kw OR suna:ti,ab,kw OR hemicrania*:ti,ab,kw) 43397

#4 #1 OR #2 OR #3 43397

#5 [mh "Botulinum Toxins"] 2594

#6 (abobotulinum:ti,ab,kw OR abobotulinumtoxin*:ti,ab,kw OR alluzience:ti,ab,kw OR azzalure:ti,ab,kw OR bocouture:ti,ab,kw OR boe-tox:ti,ab,kw OR "bont a":ti,ab,kw OR "bont serotype a":ti,ab,kw OR botox:ti,ab,kw OR botulin:ti,ab,kw OR botulinium:ti,ab,kw OR botulinum:ti,ab,kw OR BoNT-Aa:ti,ab,kw OR BoNT-A:ti,ab,kw OR cunox:ti,ab,kw OR daxibotulinum:ti,ab,kw OR daxibotulinumtoxin*:ti,ab,kw OR daxxify:ti,ab,kw OR dysport:ti,ab,kw OR evabotulinum:ti,ab,kw OR evabotulinumtoxin*:ti,ab,kw OR evosyal:ti,ab,kw OR gemibotulinum*:ti,ab,kw OR hutox:ti,ab,kw OR incobotulinum:ti,ab,kw OR incobotulinumtoxin*:ti,ab,kw OR jeuveau:ti,ab,kw OR letibotulinum:ti,ab,kw OR letibotulinumtoxin*:ti,ab,kw OR letybo:ti,ab,kw OR liztox:ti,ab,kw OR meditoxin:ti,ab,kw OR nabota:ti,ab,kw OR neuronox:ti,ab,kw OR nivobotulinum:ti,ab,kw OR nivobotulinumtoxin*:ti,ab,kw OR nuceiva:ti,ab,kw OR oculinum:ti,ab,kw OR onabotulinum:ti,ab,kw OR onabotulinumtoxin*:ti,ab,kw OR onaclostox:ti,ab,kw OR prabotulinum:ti,ab,kw OR prabotulinumtoxin*:ti,ab,kw OR

prosigne:ti,ab,kw OR purtox:ti,ab,kw OR relabotulinum:ti,ab,kw OR relabotulinumtoxin*:ti,ab,kw OR reloxin:ti,ab,kw OR vistabel:ti,ab,kw OR vistabex:ti,ab,kw OR xeomeen:ti,ab,kw OR xeomin:ti,ab,kw) 6019

#7 #5 OR #6 6019

#8 #4 AND #7 601

#9 [mh "Migraine disorders"] 3904

#10 migraine*:ti,ab,kw 10132

#11 #9 OR #10 10132

#12 (prevent*:ti,ab,kw OR treatment*:ti,ab,kw OR therapy:ti,ab,kw OR therapies:ti,ab,kw) 1397327

#13 MeSH descriptor: [undefined] explode all trees and with qualifier(s): [prevention & control - PC, drug therapy - DT] 392949

#14 #12 OR #13 1397327

#15 #11 AND #14 8693

#16 #15 NOT #8 8327

Limit to reviews: 61

4. Web of Science Core Collection

Interface: **Clarivate Analytics**

Editions and content coverage years= A&HCI - 1975 , ESCI -2019 , SCI-EXPANDED - 1945 , SSCI - 1945

Date of Search: 20 August 2024

Number of hits: 2,257

Field labels

- TS/Topic = title, abstract, author keywords and Keywords Plus
- TI= title
- AB = abstract
- AK = author keywords
- NEAR/x = within x words, regardless of order
- * = truncation of word for alternate endings
- # = 0-1 letter/number
- ? = 1 letter/number

Note: the *Exact search*-function was used for all the searches

#	Search Query	Results
1	TS=(migraine* OR headache* OR cephalalgia* OR cephalgia* OR sunct* OR suna OR hemicrania*)	140470
2	TS=(abobotulinum OR abobotulinumtoxin* OR alluzience OR azzalure OR bocouture OR boe-tox OR "bont a" OR "bont serotype a" OR botox OR botulin OR botulinium OR botulinum OR BoNT-Aa OR BoNT-A OR cunox OR daxibotulinum OR daxibotulinumtoxin* OR daxxify OR dysport OR evabotulinum OR evabotulinumtoxin* OR evosyal OR gemibotulinum* OR hutox OR incobotulinum OR incobotulinumtoxin* OR jeuveau OR letibotulinum OR letibotulinumtoxin* OR letybo OR liztox OR meditoxin OR nabota OR neuronox OR nivobotulinum OR nivobotulinumtoxin* OR nuceiva OR oculinum OR onabotulinum OR onabotulinumtoxin* OR onaclostox OR prabotulinum OR prabotulinumtoxin* OR prosigne OR purtox OR relabotulinum OR relabotulinumtoxin* OR reloxin OR vistabel OR vistabex OR xeomeen OR xeomin)	38928
3	#2 AND #1	2435
4	TS=migraine*	61040

5	TS=(prevent* OR treatment* OR therapy OR therapies)	9829544
6	TI=((systemati* OR umbrella) NEAR/3 review)	318557
7	TI=(meta-analy* OR metaanaly* OR metasynthe* OR meta-synthe*)	257643
8	#7 OR #6	444768
9	#8 AND #5 AND #4	873
10	#9 OR #3	3242
11	#9 OR #3 and English or Spanish or Portuguese (Languages)	3158
12	#9 OR #3 and English or Spanish or Portuguese (Languages) and Meeting Abstract or Editorial Material or Letter (Exclude – Document Types)	2257

5. Cinahl

Interface: **EBSCOhost** - conctent coverage from 1981

Date of Search: 20 August 2024

Number of hits: 1,381

Field labels

- MH+ = exploded Cinahl Heading
- MH = non exploded Cinahl Heading
- TI = title
- AB = abstract
- Nx = within x words, regardless of order
- * = truncation of word for alternate endings
- ? = 0-1 letter/number
- # = 1 letter/number

#	**Query**	**Results**
S18	S7 OR S17<br>Limiters - Language: Danish, English, Greek, Norwegian, Portuguese, Spanish, Swedish	1,381
S17	S10 AND S13 AND S16	580
S16	S14 OR S15	245,648
S15	PT ("systematic review" OR "Meta Analysis" OR "Meta Synthesis")	178,742
S14	TI (((systemati* OR umbrella) N3 review)) OR TI ((meta-analy* OR metaanaly* OR metasynthe* OR meta-synthe*))	168,829
S13	S11 OR S12	2,353,433
S12	MW "PC" OR MW "DT"	1,197,628
S11	TI ((prevent* OR treatment* OR therapy OR therapies)) OR AB ((prevent* OR treatment* OR therapy OR therapies))	1,687,550
S10	S8 OR S9	20,757
S9	TI migraine* OR AB migraine*	17,510
S8	(MH "Migraine")	16,082
S7	S3 AND S6	843

S6	S4 OR S5	7,965
S5	TI ((abobotulinum OR abobotulinumtoxin* OR alluzience OR azzalure OR bocouture OR boe-tox OR "bont a" OR "bont serotype a" OR botox OR botulin OR botulinium OR botulinum OR BoNT-Aa OR BoNT-A OR cunox OR daxibotulinum OR daxibotulinumtoxin* OR daxxify OR dysport OR evabotulinum OR evabotulinumtoxin* OR evosyal OR gemibotulinum* OR hutox OR incobotulinum OR incobotulinumtoxin* OR jeuveau OR letibotulinum OR letibotulinumtoxin* OR letybo OR liztox OR meditoxin OR nabota OR neuronox OR nivobotulinum OR nivobotulinumtoxin* OR nuceiva OR oculinum OR onabotulinum OR onabotulinumtoxin* OR onaclostox OR prabotulinum OR prabotulinumtoxin* OR prosigne OR purtox OR relabotulinum OR relabotulinumtoxin* OR reloxin OR vistabel OR vistabex OR xeomeen OR xeomin)) OR AB ((abobotulinum OR abobotulinumtoxin* OR alluzience OR azzalure OR bocouture OR boe-tox OR "bont a" OR "bont serotype a" OR botox OR botulin OR botulinium OR botulinum OR BoNT-Aa OR BoNT-A OR cunox OR daxibotulinum OR daxibotulinumtoxin* OR daxxify OR dysport OR evabotulinum OR evabotulinumtoxin* OR evosyal OR gemibotulinum* OR hutox OR incobotulinum OR incobotulinumtoxin* OR jeuveau OR letibotulinum OR letibotulinumtoxin* OR letybo OR liztox OR meditoxin OR nabota OR neuronox OR nivobotulinum OR nivobotulinumtoxin* OR nuceiva OR oculinum OR onabotulinum OR onabotulinumtoxin* OR onaclostox OR prabotulinum OR prabotulinumtoxin* OR prosigne OR purtox OR relabotulinum OR relabotulinumtoxin* OR reloxin OR vistabel OR vistabex OR xeomeen OR xeomin))	6,321
S4	(MH "Botulinum Toxins")	6,012
S3	S1 OR S2	48,242
S2	TI ((migraine* OR headache* OR cephalalgia* OR cephalgia* OR sunct* OR suna OR hemicrania*)) OR AB ((migraine* OR headache* OR cephalalgia* OR cephalgia* OR sunct* OR suna OR hemicrania*))	41,319
S1	(MH "Headache+")	31,871
